# Supplementary material for: THOC1 deficiency leads to late-onset nonsyndromic hearing loss through p53-mediated hair cell apoptosis
Source: PLoS Genet. 2020 Aug 10;16(8):e1008953. doi: 10.1371/journal.pgen.1008953 (PMC7444544; doi:10.1371/journal.pgen.1008953)
Supplement: S1 Fig — Hearing thresholds were averages of both ears. All affected members exhibited symmetric audiometric configuration. (PDF) [file pgen.1008953.s001.pdf]

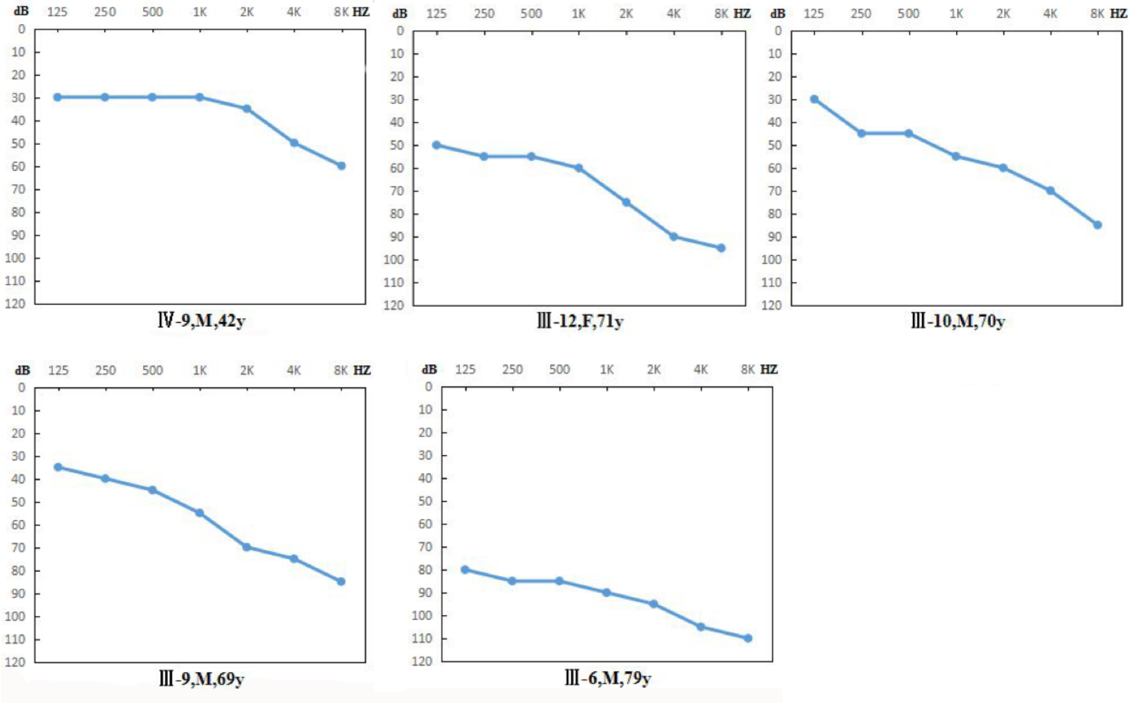

S1 Fig. Audiograms of Family SH. Hearing thresholds were averages of both ears. All affected members exhibited symmetric audiometric configuration.
