# Supplementary material for: THOC1 deficiency leads to late-onset nonsyndromic hearing loss through p53-mediated hair cell apoptosis
Source: PLoS Genet. 2020 Aug 10;16(8):e1008953. doi: 10.1371/journal.pgen.1008953 (PMC7444544; doi:10.1371/journal.pgen.1008953)
Supplement: S2 Fig — Relative expression level is shown as the ratios of that of the affected individual III-10 over the unaffected individual IV-6. None of the 11 genes show significant (P>0.05, t-test) differential expression in the affected individual in comparison with that of the internal control gene (GAPDH). (PDF) [file pgen.1008953.s002.pdf]

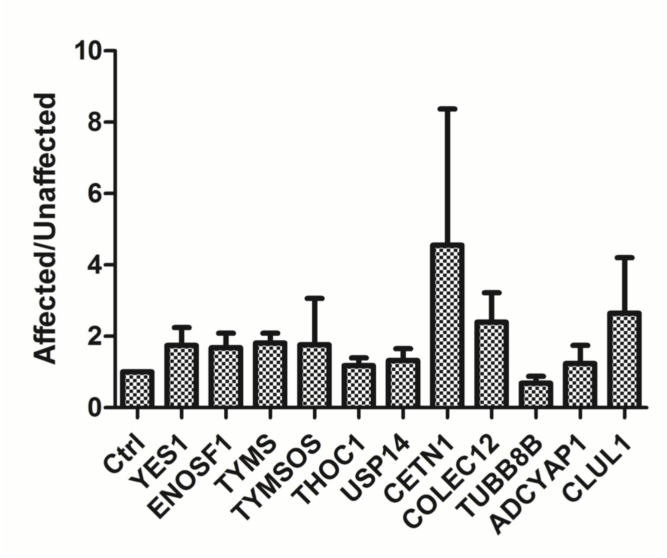

S2 Fig. Quantitative reversed-transcript PCR of 11 protein-coding genes in the critical interval utilizing blood from two family members with (III-10) and without (IV-6) the c.547C>G mutation. Relative expression level is shown as the ratios of that of the affected individual III-10 over the unaffected individual IV-6. None of the 11 genes show significant (*P*>0.05, t-test) differential expression in the affected individual in comparison with that of the internal control gene (*GAPDH*).
