# Supplementary material for: THOC1 deficiency leads to late-onset nonsyndromic hearing loss through p53-mediated hair cell apoptosis
Source: PLoS Genet. 2020 Aug 10;16(8):e1008953. doi: 10.1371/journal.pgen.1008953 (PMC7444544; doi:10.1371/journal.pgen.1008953)
Supplement: S3 Fig — (a) Primer design that amplifies multiple exons (4–10) containing and flanking the c.547C>G mutation (red dash) in exon 7. (b) No differential mRNA expression or alternatively spliced transcript can be detected in the affected individual III-10. (c) Sequencing results of the amplicons. (PDF) [file pgen.1008953.s003.pdf]

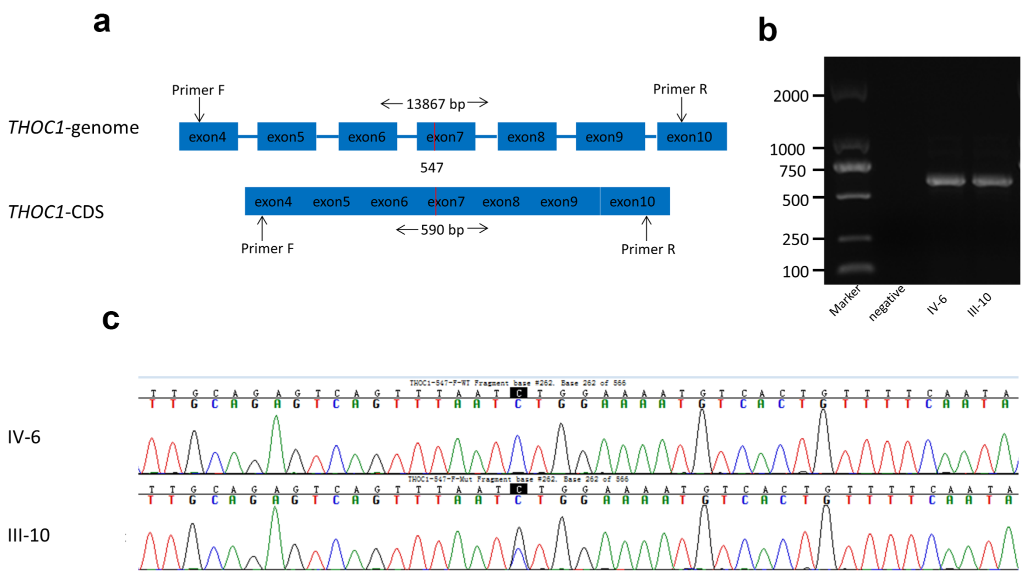

S3 Fig. Reversed-transcript PCR of exons 4-10 of *THOC1* in individuals III-10 and IV-6. (a) Primer design that amplifies multiple exons (4-10) containing and flanking the c.547C>G mutation (red dash) in exon 7. (b) No differential mRNA expression or alternatively spliced transcript can be detected in the affected individual III-10. (c) Sequencing results of the amplicons.
