# Supplementary material for: THOC1 deficiency leads to late-onset nonsyndromic hearing loss through p53-mediated hair cell apoptosis
Source: PLoS Genet. 2020 Aug 10;16(8):e1008953. doi: 10.1371/journal.pgen.1008953 (PMC7444544; doi:10.1371/journal.pgen.1008953)
Supplement: S5 Fig — (a) Western blot analysis of THOC1 expression in 293t cells and THOC1 knockout 293t cells. (b) Realtime PCR analysis of THOC1 expression in several cell lines. (PDF) [file pgen.1008953.s005.pdf]

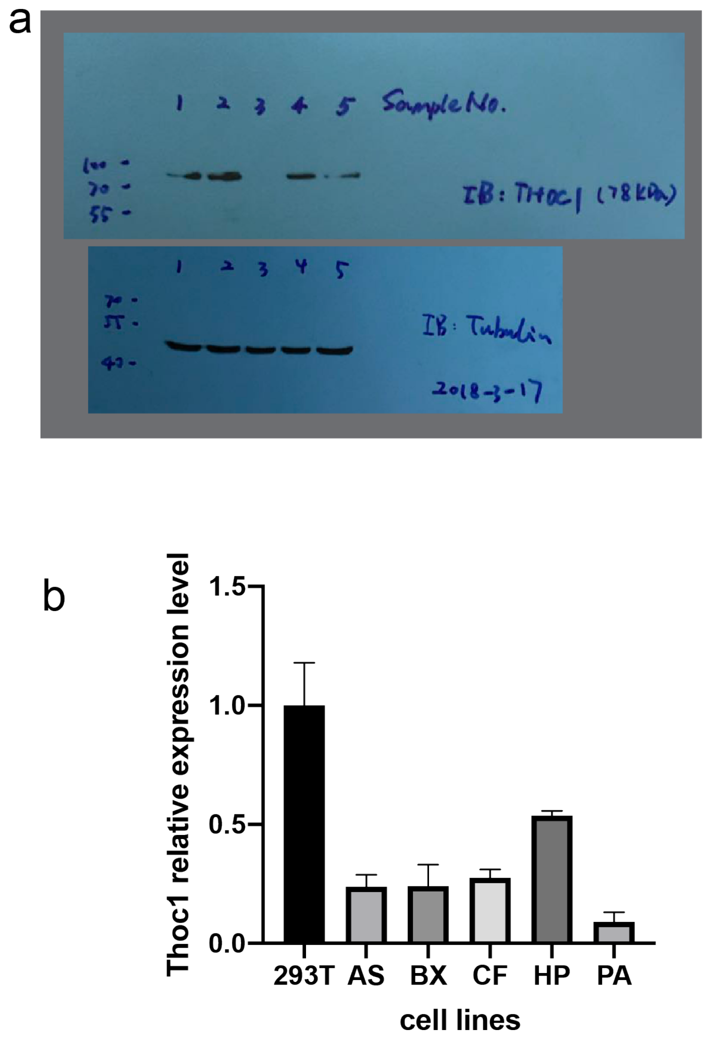

S5 Fig. The validation of monoclonal Anti-THOC1 antibody. (a) Western blot analysis of THOC1 expression in 293t cells and THOC1 knockout 293t cells. (b) Realtime PCR analysis of *THOC1* expression in several cell lines.
