# Supplementary material for: THOC1 deficiency leads to late-onset nonsyndromic hearing loss through p53-mediated hair cell apoptosis
Source: PLoS Genet. 2020 Aug 10;16(8):e1008953. doi: 10.1371/journal.pgen.1008953 (PMC7444544; doi:10.1371/journal.pgen.1008953)
Supplement: S6 Fig — Confocal microscopic imaging analysis of THOC1 antibody staining in P0 mouse. Blue: DAPI staining of the cell nuclei. Red: Myosin 7a staining marking hair cells. Green: THOC1 antibody staining. Bars, 40 μm. (PDF) [file pgen.1008953.s006.pdf]

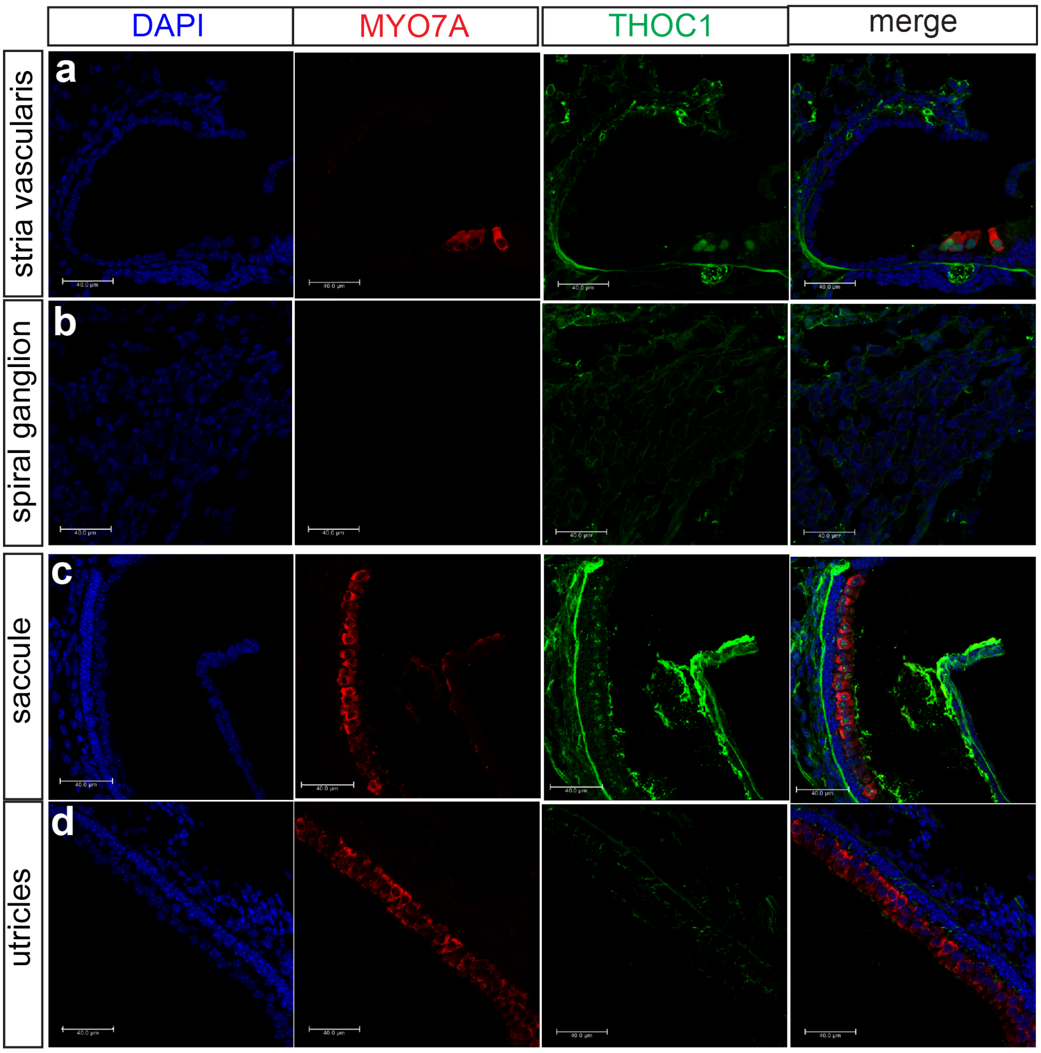

S6 Fig. The expression of THOC1 in mouse auditory organ. Confocal microscopic imaging analysis of THOC1 antibody staining in P0 mouse. Blue: DAPI staining of the cell nuclei. Red: Myosin 7a staining marking hair cells. Green: THOC1 antibody staining. Bars, 40 μm.
