# Supplementary material for: THOC1 deficiency leads to late-onset nonsyndromic hearing loss through p53-mediated hair cell apoptosis
Source: PLoS Genet. 2020 Aug 10;16(8):e1008953. doi: 10.1371/journal.pgen.1008953 (PMC7444544; doi:10.1371/journal.pgen.1008953)
Supplement: S7 Fig — (a) The targeting sites of gRNAs. (b) Mutation pattern of gRNA1/gRNA2 and cas9 mRNA coinjected embryos. Numbers in the brackets show the number of nucleotides were deleted (-) or inserted (+). Inserted nucleotide is in red. WT, wild type. (PDF) [file pgen.1008953.s007.pdf]

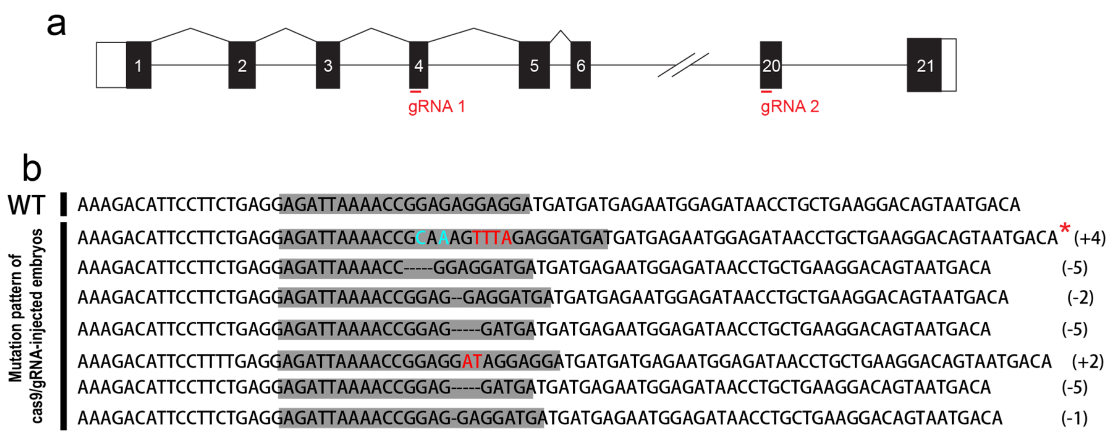

S7 Fig. *Thoc1* gene knockout using CRISPR/Cas9 system.

(a) The targeting sites of gRNAs. (b) Mutation pattern of gRNA1/gRNA2 and cas9 mRNA coinjected embryos. Numbers in the brackets show the number of nucleotides were deleted (-) or inserted (+). Inserted nucleotide is in red. WT, wild type.
