# Supplementary material for: THOC1 deficiency leads to late-onset nonsyndromic hearing loss through p53-mediated hair cell apoptosis
Source: PLoS Genet. 2020 Aug 10;16(8):e1008953. doi: 10.1371/journal.pgen.1008953 (PMC7444544; doi:10.1371/journal.pgen.1008953)
Supplement: S8 Fig — (a) The fluorescence microscopic imaging analysis of control and thoc1 mutants Tg(cldnb:lynGFP) zebrafish embryos at 48 hpf. (b) The neuromasts detected by whole mount in situ hybridization analysis of eya1 at 48 hpf. (c) The fluorescence microscopic imaging analysis of control and thoc1 mutants Tg(cldnb:lynGFP) zebrafish embryos at 3 dpf. (d) The statistical analysis of the number of neuromasts at each side of the control (n = 12) and thoc1 mutant (n = 24) embryo trunk at 48 hpf. t-test, ****P < 0.0001. (e) The statistical analysis of the number of neuromasts at each side of the control (n = 12) and thoc1 mutant (n = 23) embryo trunk at 3 dpf. t-test, ****P < 0.0001. (PDF) [file pgen.1008953.s008.pdf]

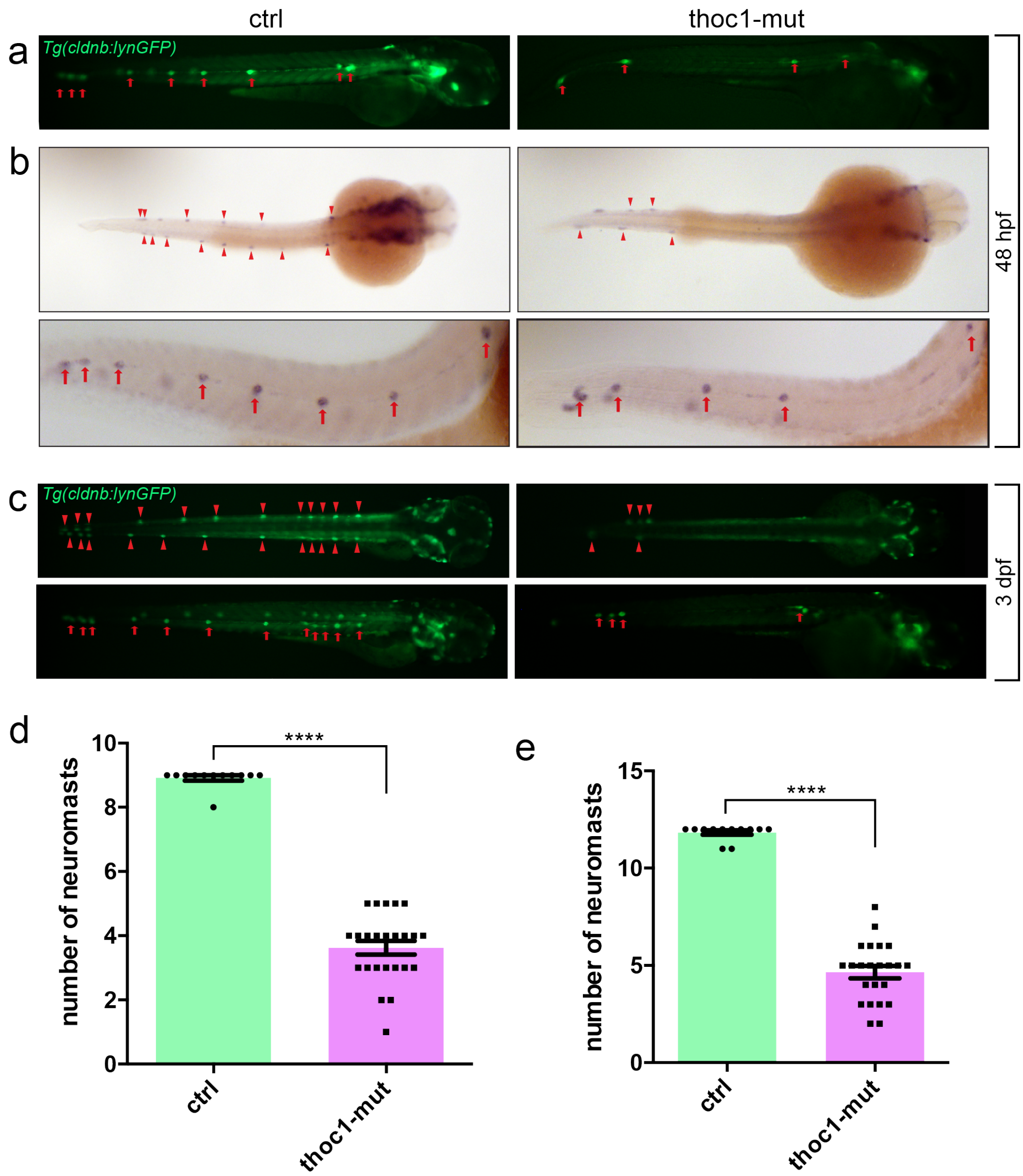

S8 Fig. *Thoc1* knockout caused the reduction of neuromasts in zebrafish. (a) The fluorescence microscopic imaging analysis of control and thoc1 mutants *Tg(cldnb:lynGFP)* zebrafish embryos at 48 hpf. (b) The neuromasts detected by whole mount *in situ* hybridization analysis of *eya1* at 48 hpf. (c) The fluorescence microscopic imaging analysis of control and thoc1 mutants *Tg(cldnb:lynGFP)* zebrafish embryos at 3 dpf. (d) The statistical analysis of the number of neuromasts at each side of the control (n=12) and *thoc1* mutant (n=24) embryo trunk at 48 hpf. t-test, ****P < 0.0001. (e) The statistical analysis of the number of neuromasts at each side of the control (n=12) and *thoc1* mutant (n=23) embryo trunk at 3 dpf. t-test, ****P < 0.0001.
