# Supplementary material for: THOC1 deficiency leads to late-onset nonsyndromic hearing loss through p53-mediated hair cell apoptosis
Source: PLoS Genet. 2020 Aug 10;16(8):e1008953. doi: 10.1371/journal.pgen.1008953 (PMC7444544; doi:10.1371/journal.pgen.1008953)
Supplement: S9 Fig — (a) Confocal microscopic imaging analysis the hair cells in otic vesicle of control and thoc1 mutants Tg(pou4f3:gap43-GFP) at 3 dpf. (b) Statistical analysis of the hair cells in otic vesicle of control and thoc1 mutants. t-test, ****, p<0.0001. (PDF) [file pgen.1008953.s009.pdf]

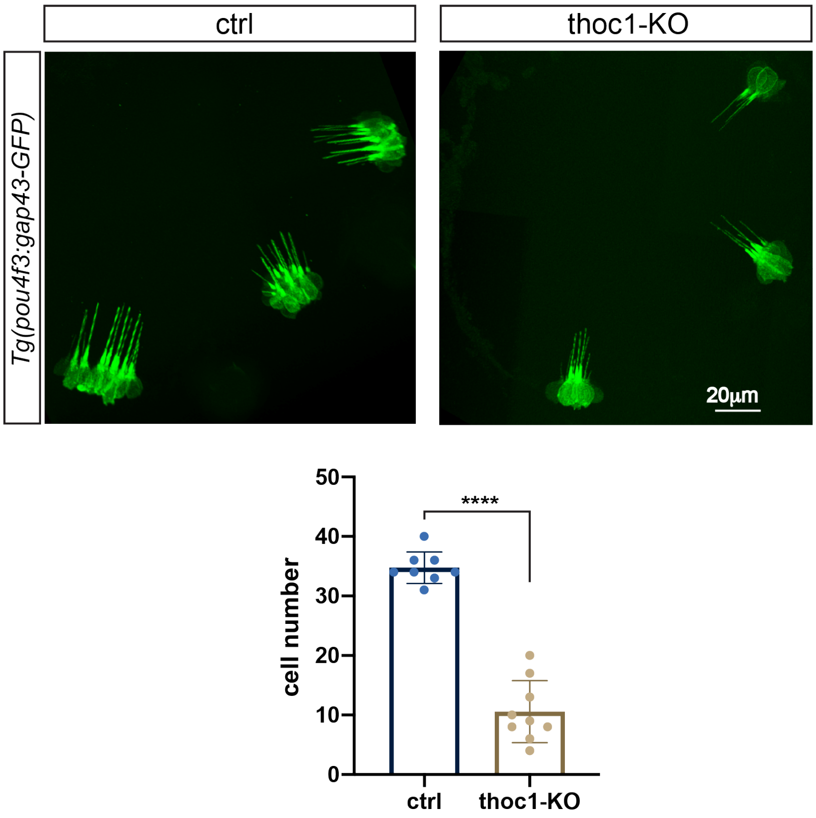

S9 Fig. *Thoc1* deficiency caused hair cell developmental defects in zebrafish. (a) Confocal microscopic imaging analysis the hair cells in otic vesicle of control and *thoc1* mutants *Tg(pou4f3:gap43-GFP)* at 3 dpf. (b) Statistical analysis of the hair cells in otic vesicle of control and *thoc1* mutants. *t*-test, ****, $p<0.0001$.
