# Supplementary material for: THOC1 deficiency leads to late-onset nonsyndromic hearing loss through p53-mediated hair cell apoptosis
Source: PLoS Genet. 2020 Aug 10;16(8):e1008953. doi: 10.1371/journal.pgen.1008953 (PMC7444544; doi:10.1371/journal.pgen.1008953)
Supplement: S13 Fig — (a) The fluorescence microscopic imaging analysis of control, p53 knockout (KO), thoc1 morphants, and p53 KO+ thoc1-MO embryos stained with FM1-43FX at 3 dpf. (b, c) Statistical analysis of the hair cell clusters in trunk and head of control, p53 knockout (KO), thoc1 morphants, and p53 KO+ thoc1-MO embryos. One-way ANOVA, ****P<0.0001, **P<0.01. (d) Confocal imaging analysis of the hair cells in control, p53 knockout (KO), thoc1 morphants, and p53 KO+ thoc1-MO embryos. (PDF) [file pgen.1008953.s013.pdf]

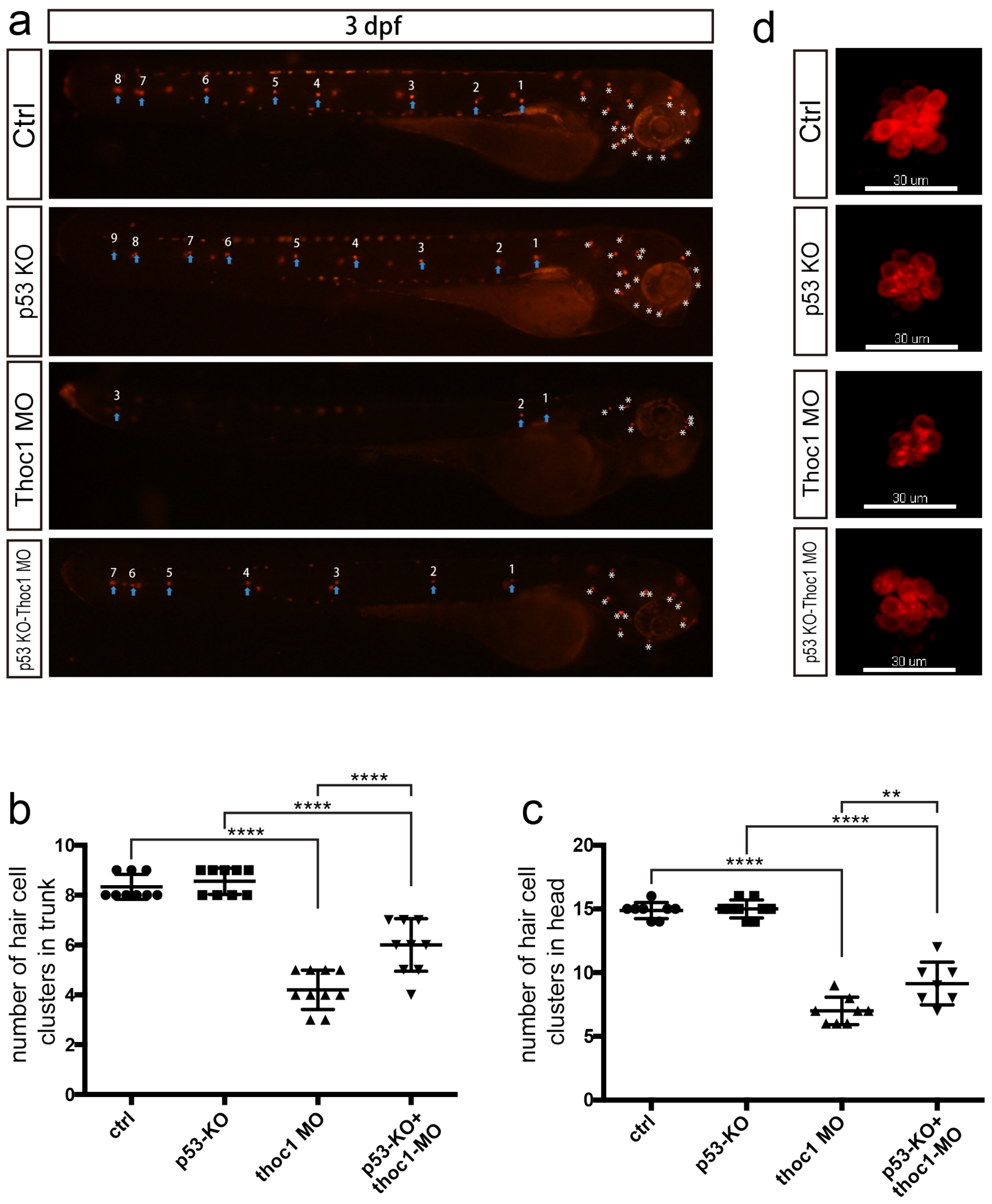

S13 Fig. P53 deficiency partially rescues the hair cell developmental defects in thoc1 morphants. (a) The fluorescence microscopic imaging analysis of control, *p53* knockout (KO), *thoc1* morphants, and *p53* KO+ *thoc1*-MO embryos stained with FM1-43FX at 3 dpf. (b, c) Statistical analysis of the hair cell clusters in trunk and head of control, *p53* knockout (KO), *thoc1* morphants, and *p53* KO+ *thoc1*-MO embryos. One-way ANOVA, *****P*<0.0001, ***P*<0.01. (d) Confocal imaging analysis of the hair cells in control, *p53* knockout (KO), *thoc1* morphants, and *p53* KO+ *thoc1*-MO embryos.
