# Supplementary material for: THOC1 deficiency leads to late-onset nonsyndromic hearing loss through p53-mediated hair cell apoptosis
Source: PLoS Genet. 2020 Aug 10;16(8):e1008953. doi: 10.1371/journal.pgen.1008953 (PMC7444544; doi:10.1371/journal.pgen.1008953)
Supplement: S14 Fig — Arrowheads indicate hair cell clusters. (PDF) [file pgen.1008953.s014.pdf]

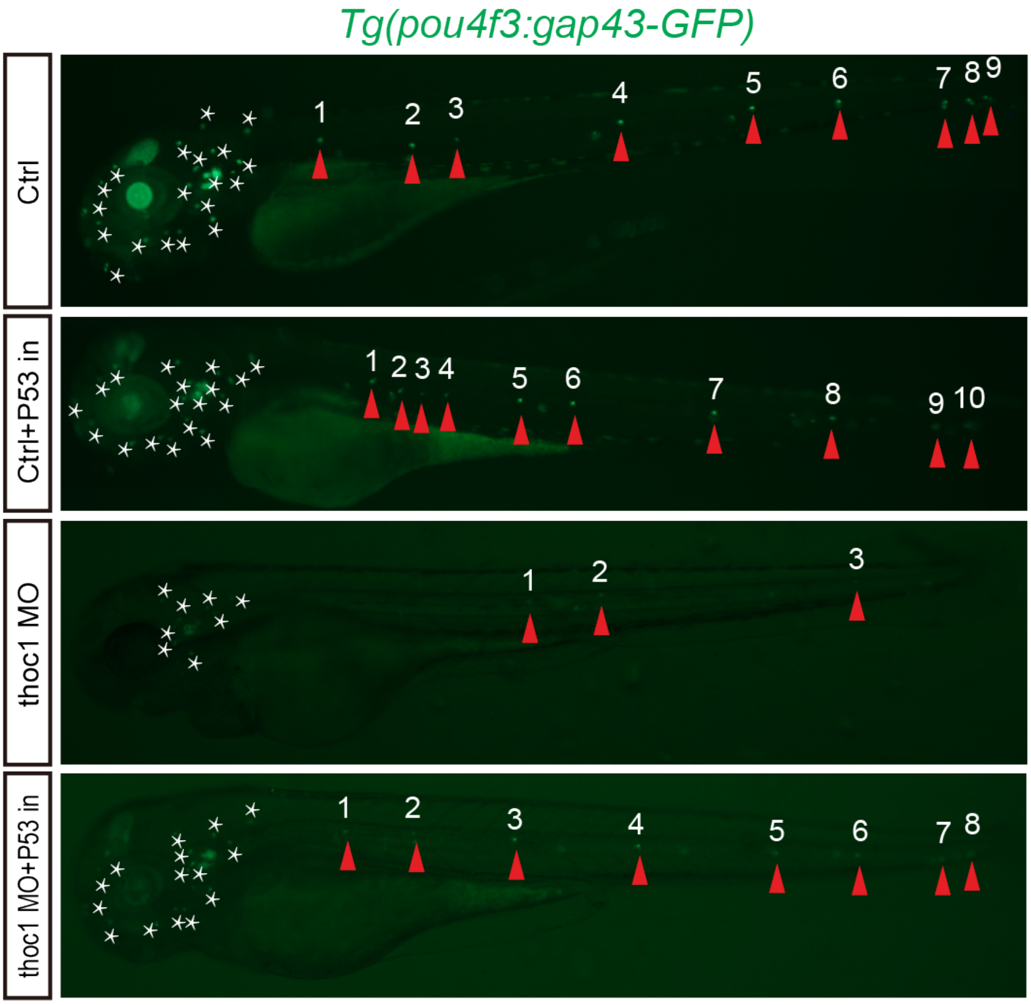

S14 Fig. Fluorescence microscopic imaging analysis of thoc1 knockout Tg(*pou4f3:gap43-GFP*) line at 3 dpf. Arrowheads indicate hair cell clusters.
