# Supplementary material for: THOC1 deficiency leads to late-onset nonsyndromic hearing loss through p53-mediated hair cell apoptosis
Source: PLoS Genet. 2020 Aug 10;16(8):e1008953. doi: 10.1371/journal.pgen.1008953 (PMC7444544; doi:10.1371/journal.pgen.1008953)
Supplement: S1 Table — (PDF) [file pgen.1008953.s015.pdf]

S1 Table. The critical interval from linage analysis of Family SH

CHR	POS (bp)	LABEL	MODEL	LOD
18	139767	rs4797697	Dominant_Model	4.8939
18	438391	rs899726	Dominant_Model	4.9292
18	592581	rs7244330	Dominant_Model	4.9318
18	675787	rs2260821	Dominant_Model	4.9332
18	854563	rs3897588	Dominant_Model	4.9341
18	908776	rs928980	Dominant_Model	4.9342
18	1046303	rs1022177	Dominant_Model	4.9342
18	1216988	rs12709528	Dominant_Model	4.9342
18	1397222	rs9953033	Dominant_Model	4.8651
18	1574368	rs11080868	Dominant_Model	1.8229
