# Supplementary material for: THOC1 deficiency leads to late-onset nonsyndromic hearing loss through p53-mediated hair cell apoptosis
Source: PLoS Genet. 2020 Aug 10;16(8):e1008953. doi: 10.1371/journal.pgen.1008953 (PMC7444544; doi:10.1371/journal.pgen.1008953)
Supplement: S2 Table — (PDF) [file pgen.1008953.s016.pdf]

S2 Table. Candidate pathogenic variants identified by exome sequencing of five members of Family SH

CHR	Pos (bp)	Gene	Variants	Segregation with phenotype
chr4	190874240	*FRG1*	NM_004477: c.C277G (p.P93A)	No
chr4	190874243	*FRG1*	NM_004477: c.C280A (p.P94T)	No
chr18	254329	*THOC1*	NM_005131: c.C547G(p.L183V)	Yes
