# Supplementary material for: THOC1 deficiency leads to late-onset nonsyndromic hearing loss through p53-mediated hair cell apoptosis
Source: PLoS Genet. 2020 Aug 10;16(8):e1008953. doi: 10.1371/journal.pgen.1008953 (PMC7444544; doi:10.1371/journal.pgen.1008953)
Supplement: S1 Data — (PDF) [file pgen.1008953.s017.pdf]

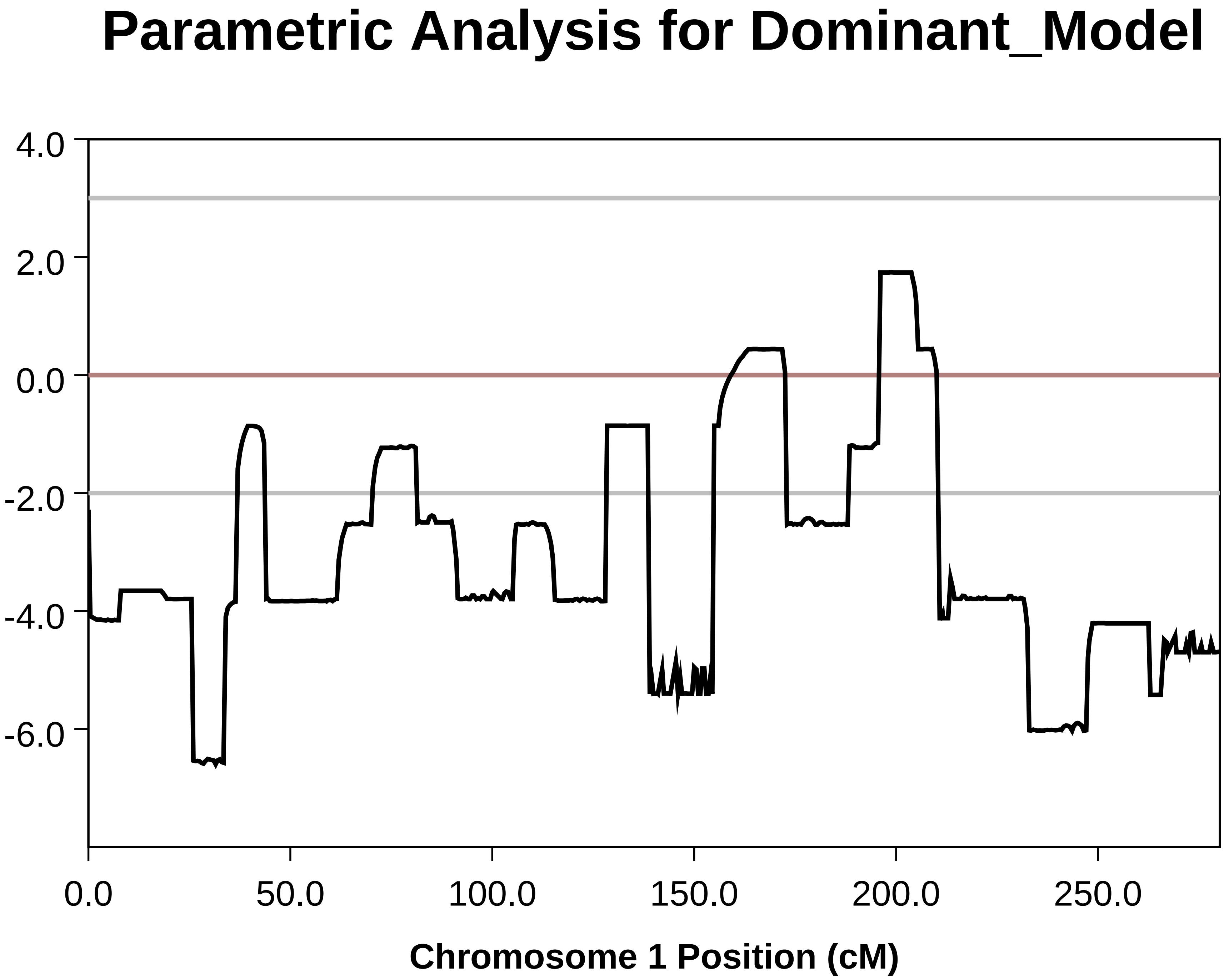
Parametric Analysis for Dominant_Model
4.0
2.0
0.0
-2.0
-4.0
-6.0
0.0
50.0
100.0
150.0
200.0
250.0
Chromosome 1 Position (cM)

Parametric Analysis for Dominant_Model

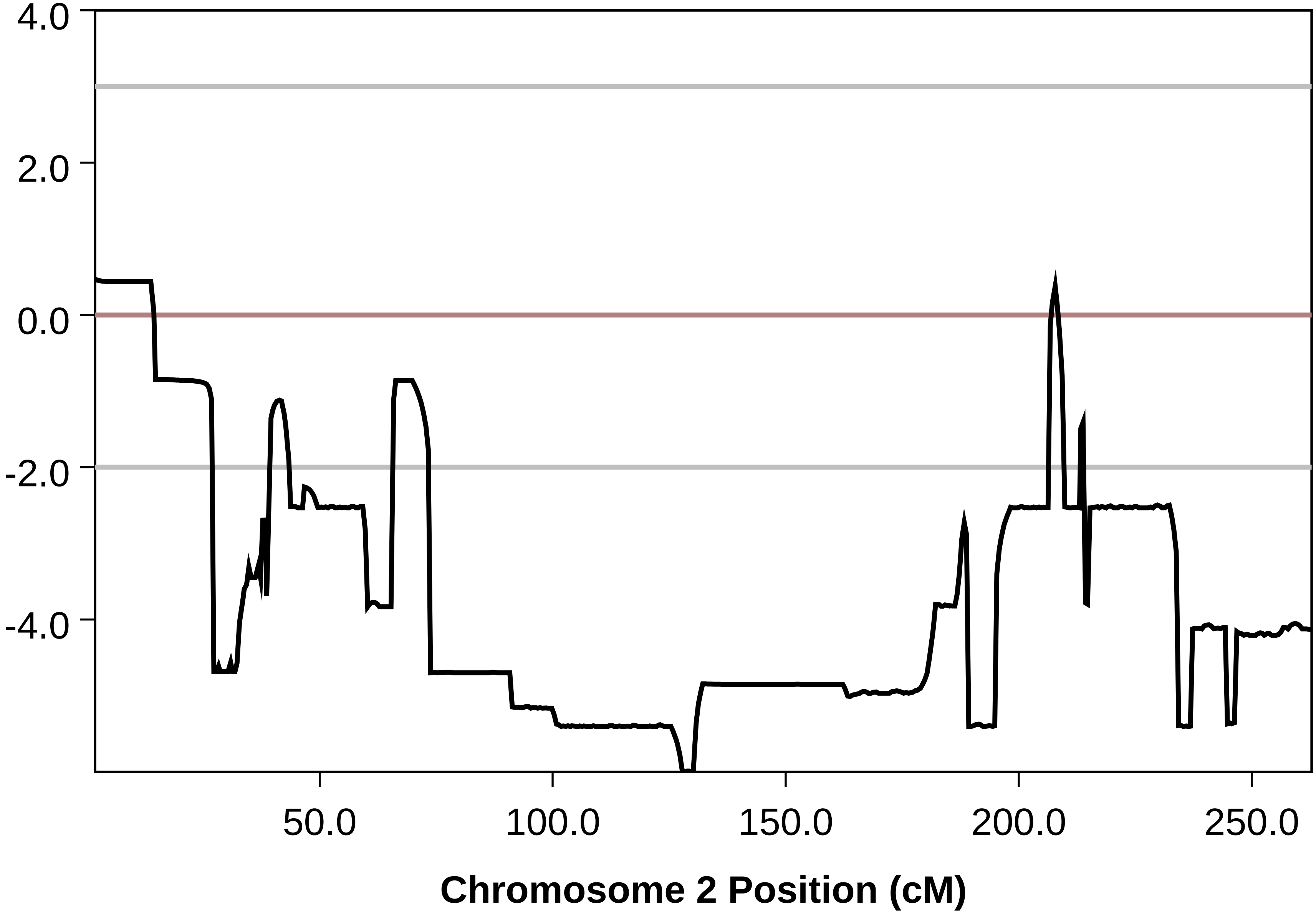

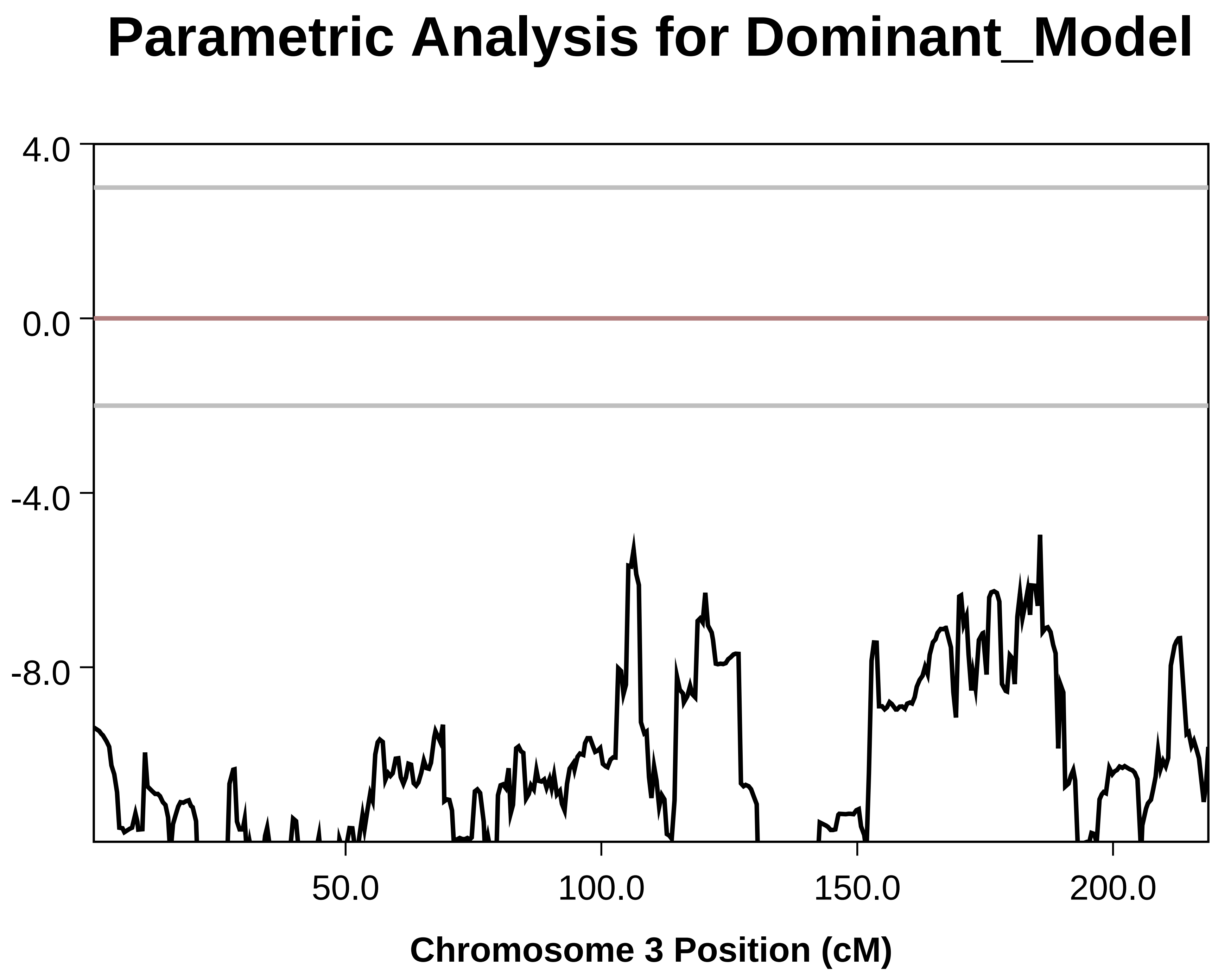
Parametric Analysis for Dominant_Model
4.0
0.0
-4.0
-8.0
50.0
100.0
150.0
200.0
Chromosome 3 Position (cM)

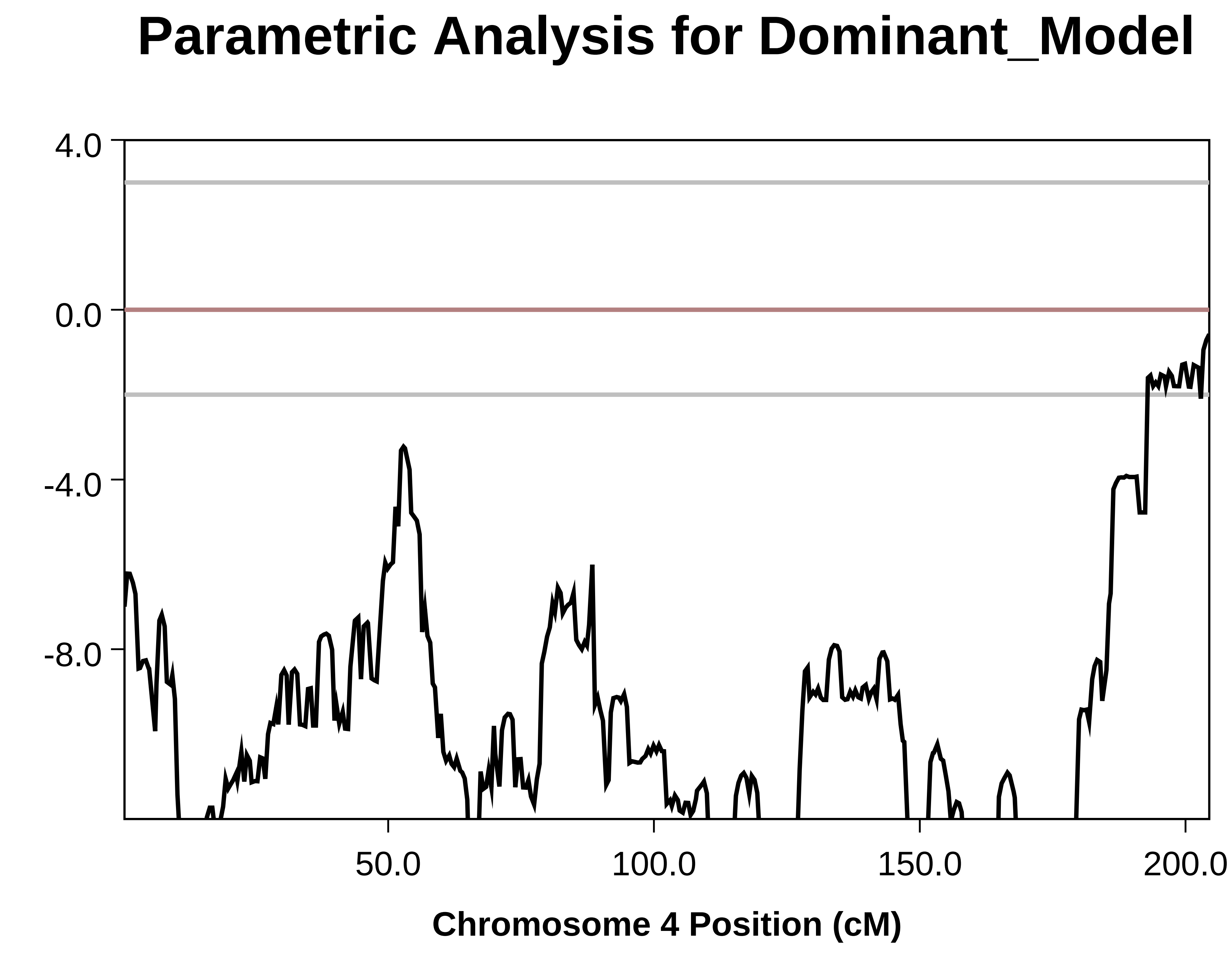
Parametric Analysis for Dominant_Model
4.0
0.0
-4.0
-8.0
50.0
100.0
150.0
200.0
Chromosome 4 Position (cM)

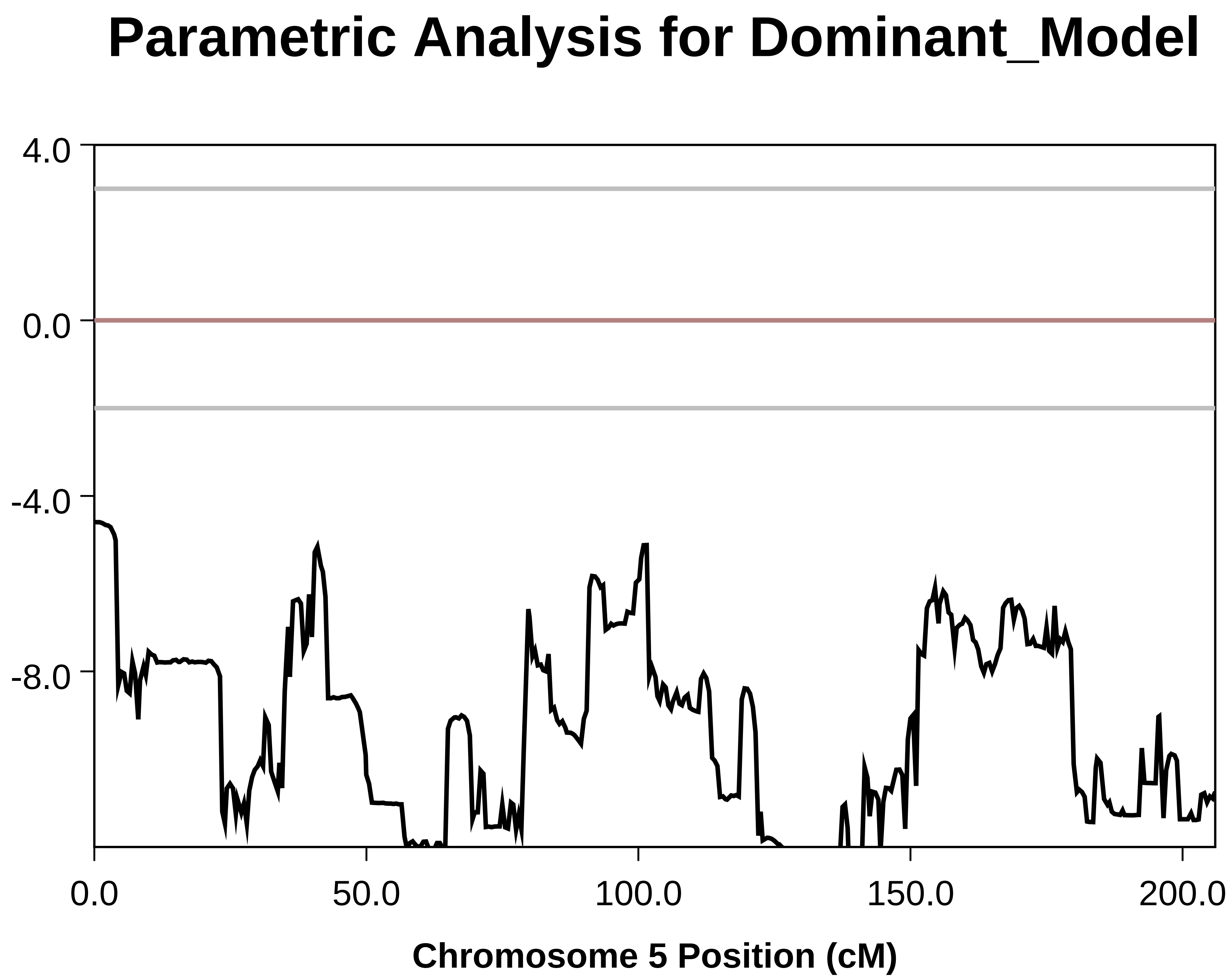
Parametric Analysis for Dominant_Model
4.0
0.0
-4.0
-8.0
0.0
50.0
100.0
150.0
200.0
Chromosome 5 Position (cM)

Parametric Analysis for Dominant_Model

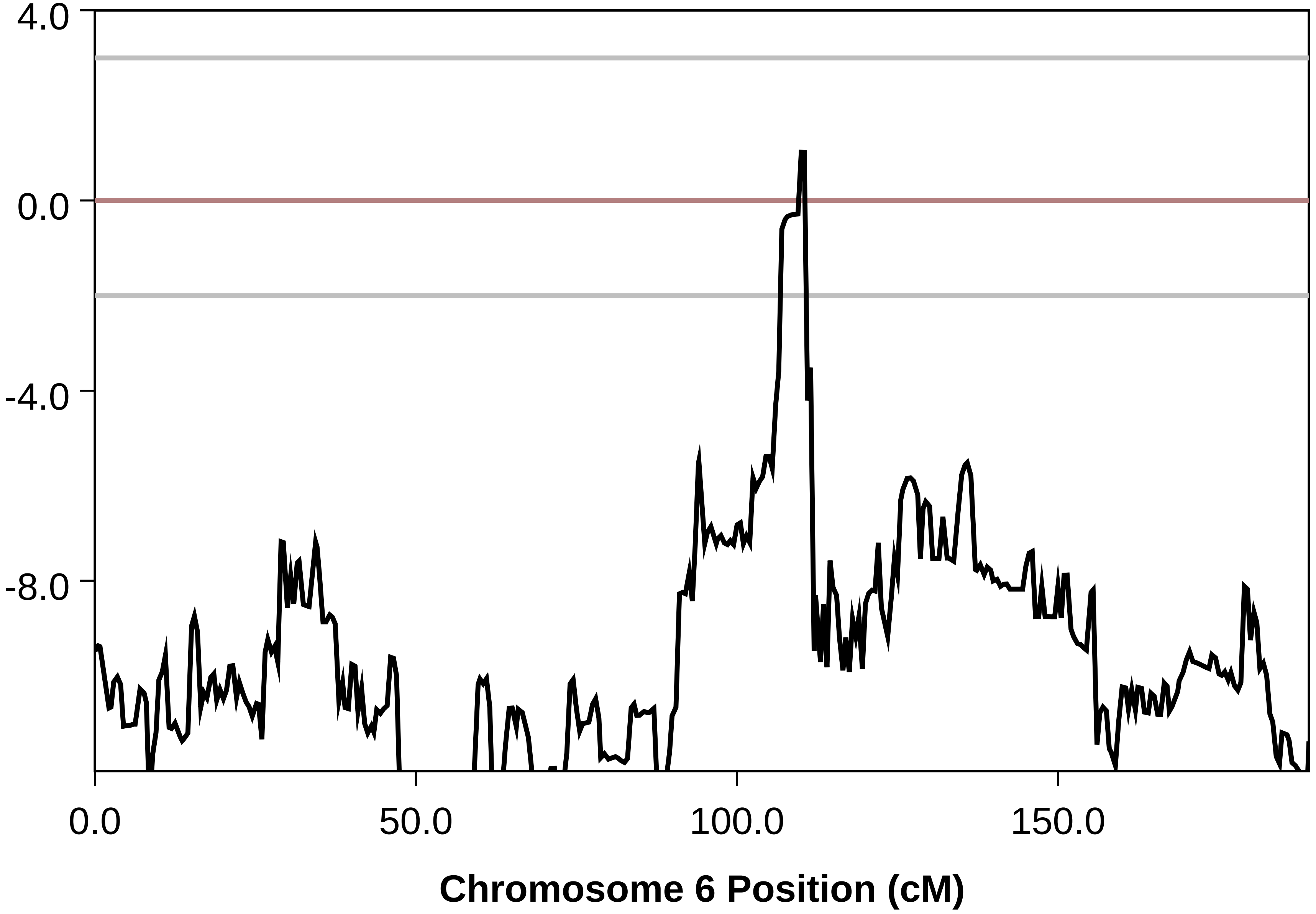

Parametric Analysis for Dominant_Model

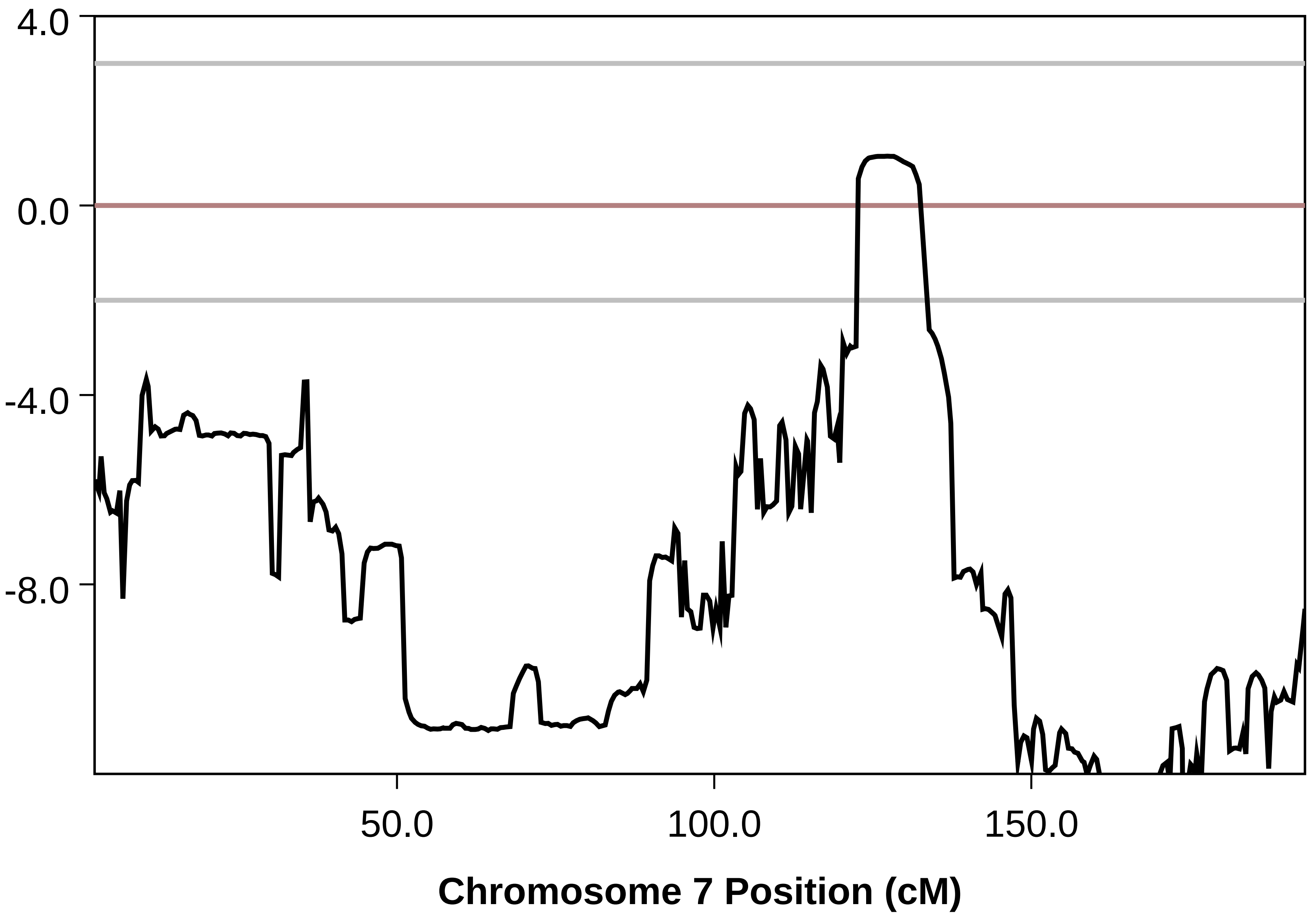

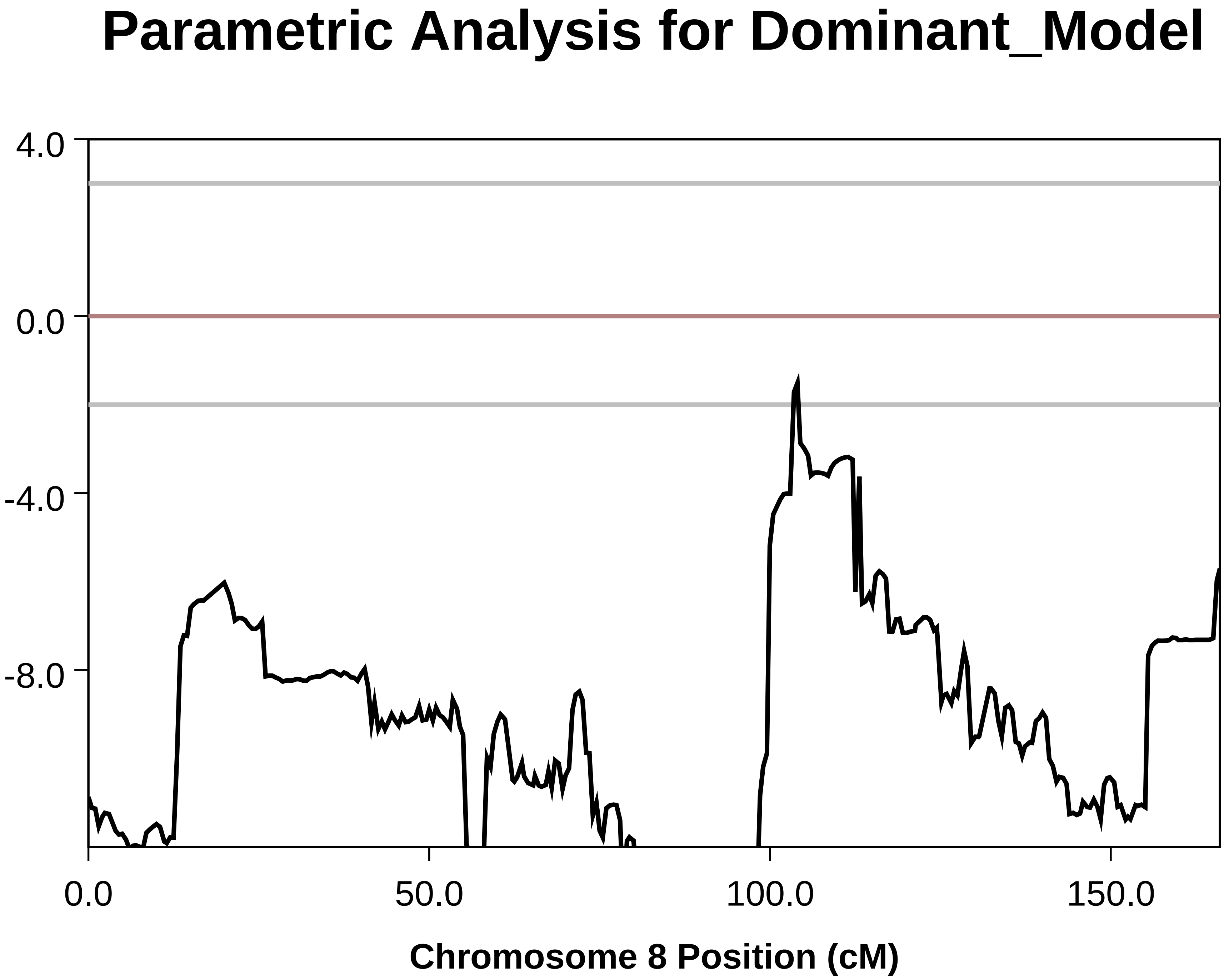

Parametric Analysis for Dominant_Model
4.0
0.0
-4.0
-8.0
0.0
50.0
100.0
150.0
Chromosome 8 Position (cM)

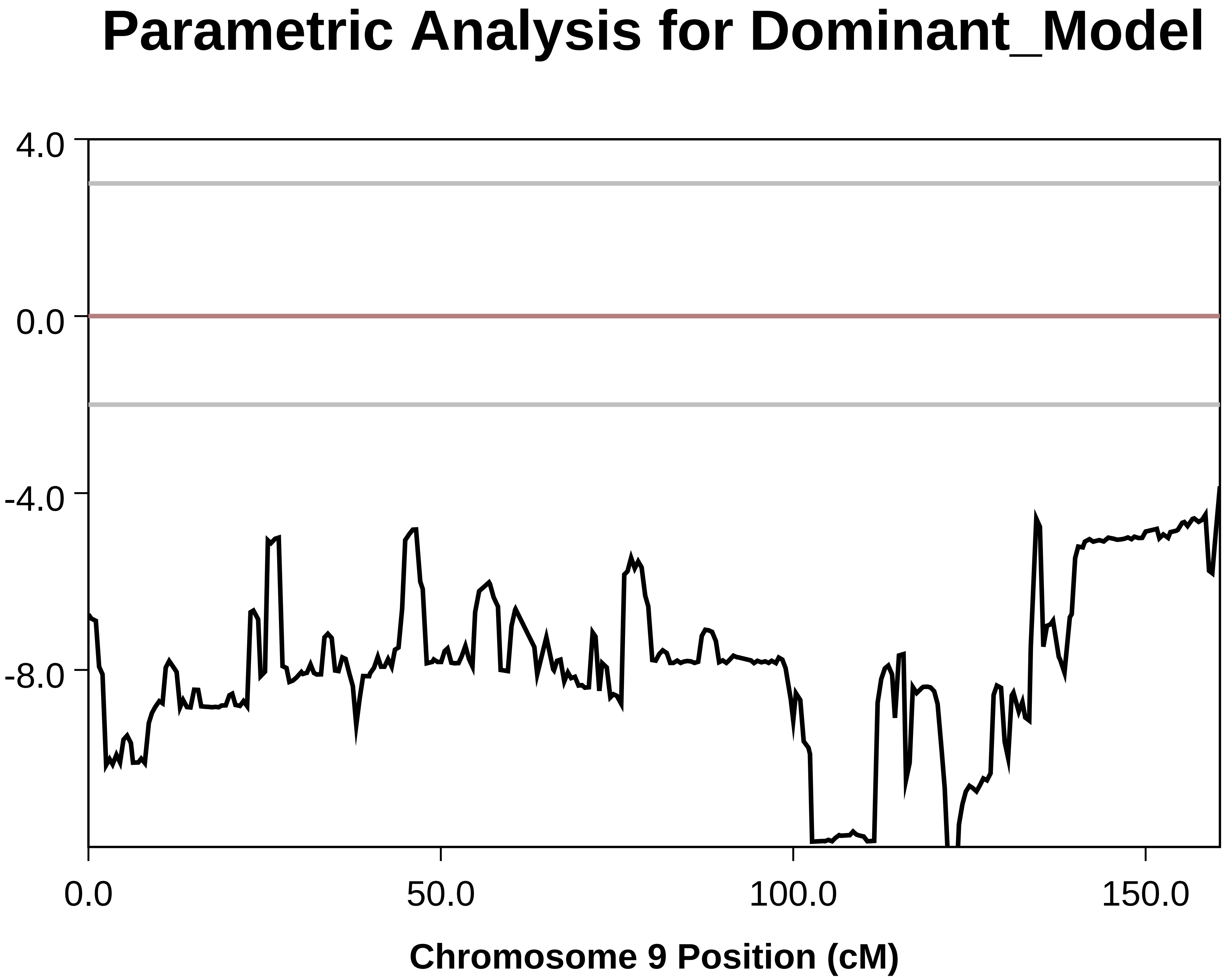
Parametric Analysis for Dominant_Model
4.0
0.0
-4.0
-8.0
0.0
50.0
100.0
150.0
Chromosome 9 Position (cM)

Parametric Analysis for Dominant_Model

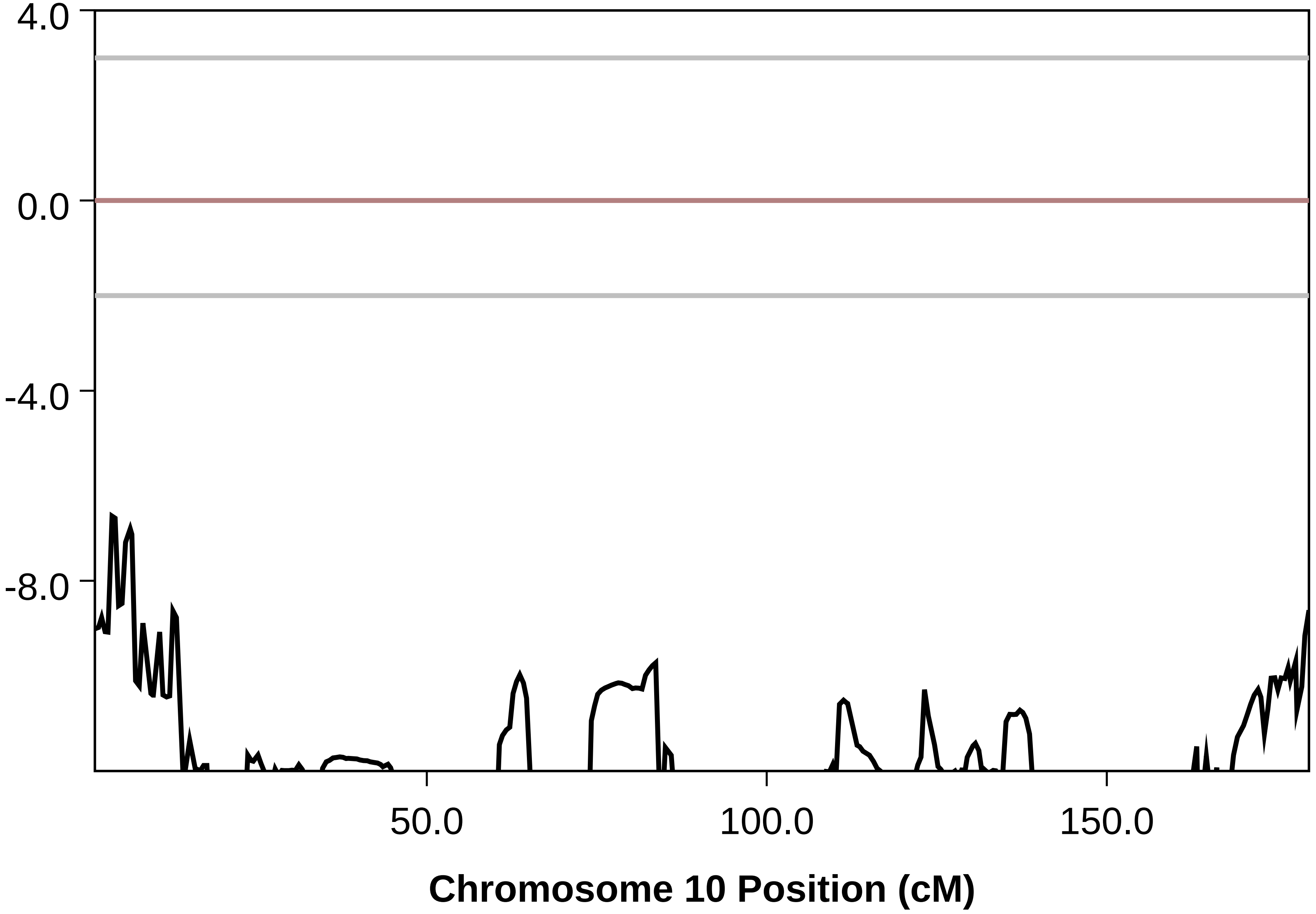

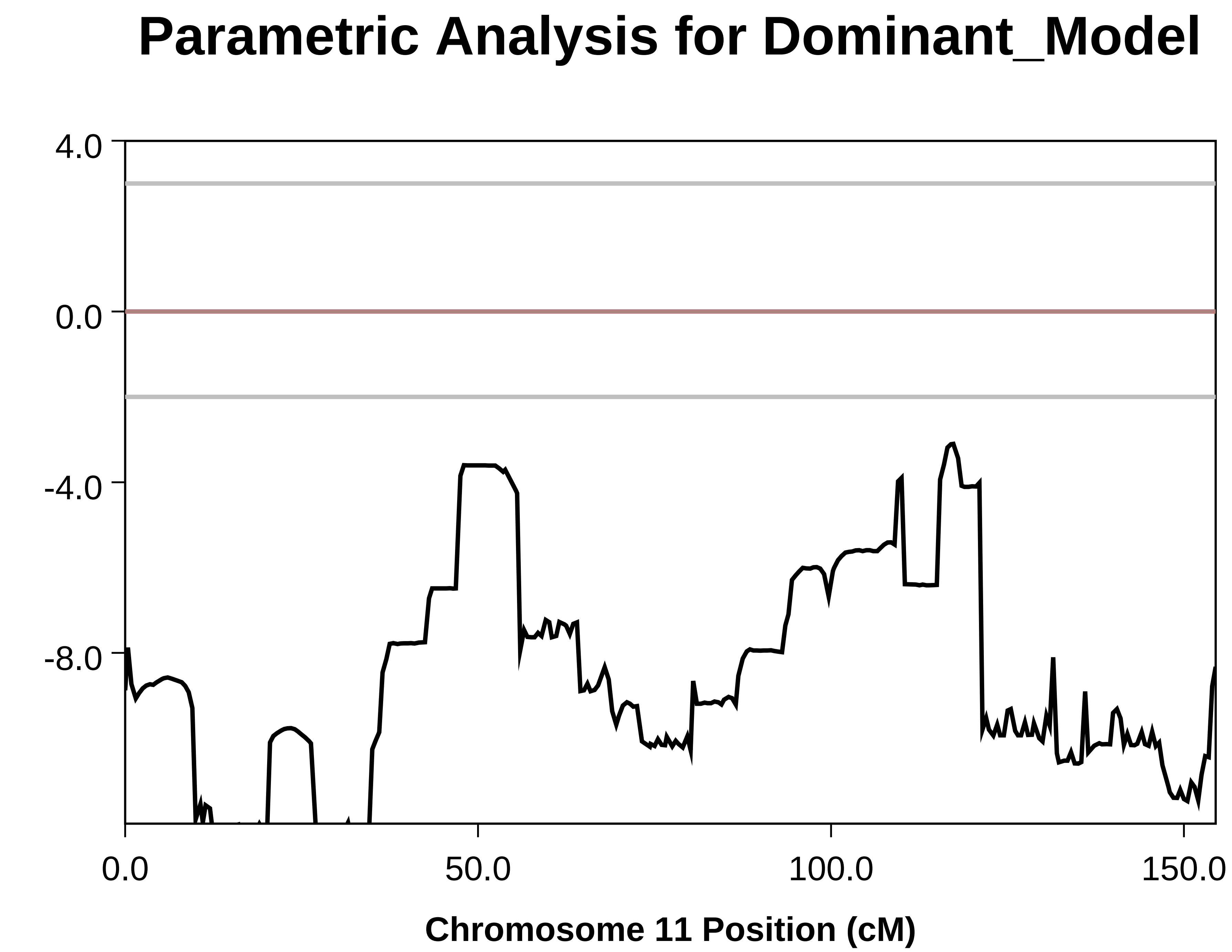

Parametric Analysis for Dominant_Model
4.0
0.0
-4.0
-8.0
0.0
50.0
100.0
150.0
Chromosome 11 Position (cM)

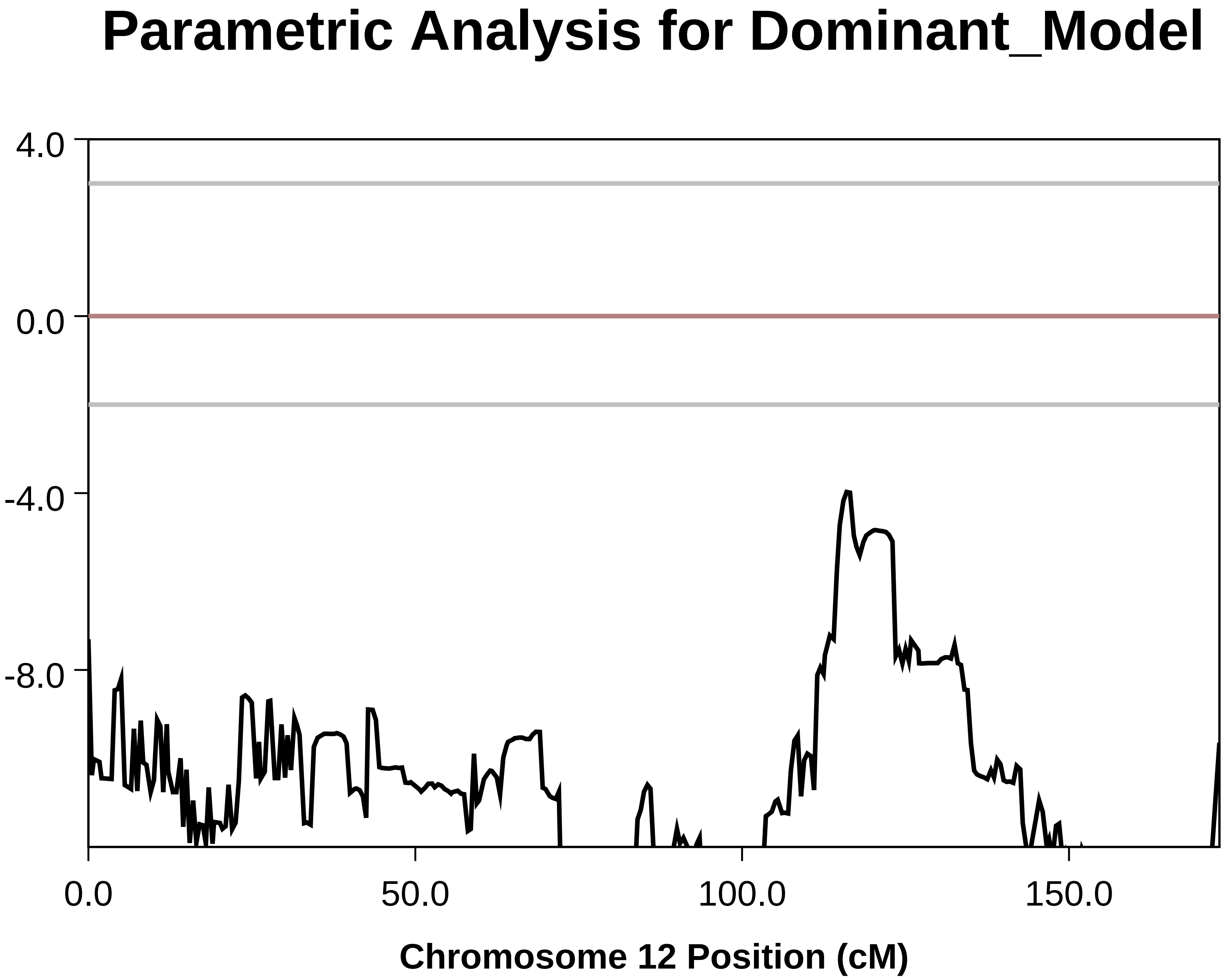

Parametric Analysis for Dominant_Model
4.0
0.0
-4.0
-8.0
0.0
50.0
100.0
150.0
Chromosome 12 Position (cM)

Parametric Analysis for Dominant_Model

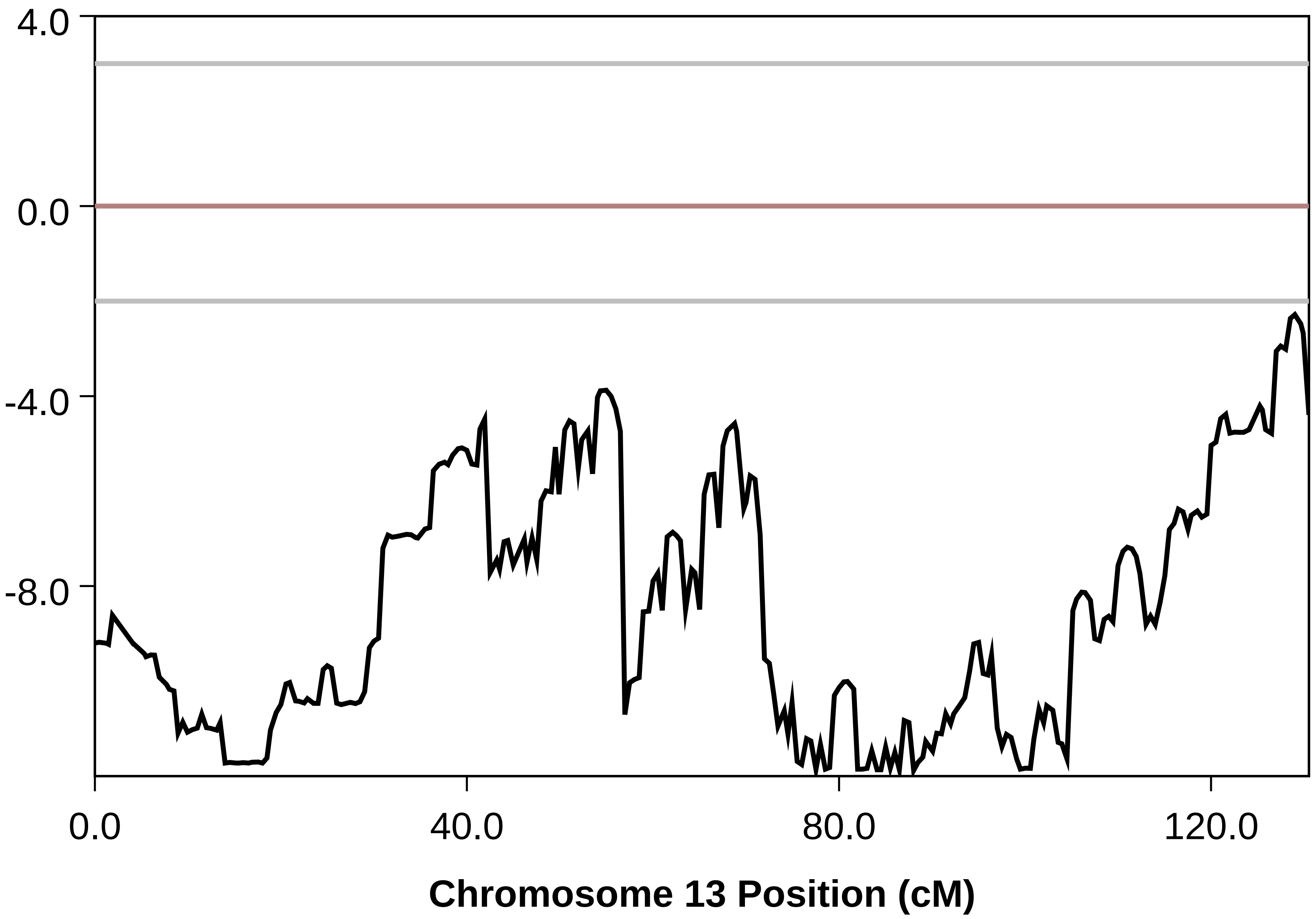

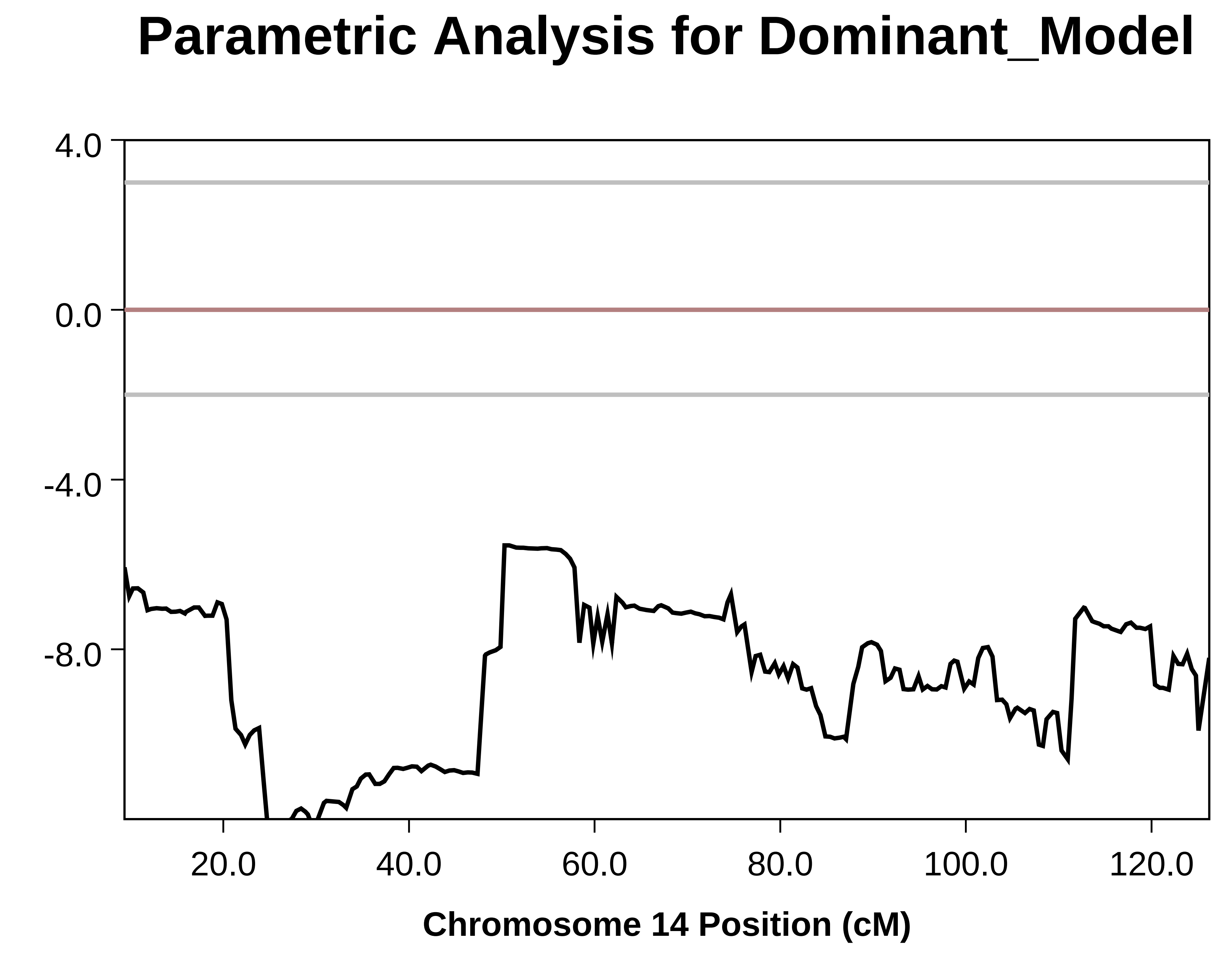
Parametric Analysis for Dominant_Model
4.0
0.0
-4.0
-8.0
20.0
40.0
60.0
80.0
100.0
120.0
Chromosome 14 Position (cM)

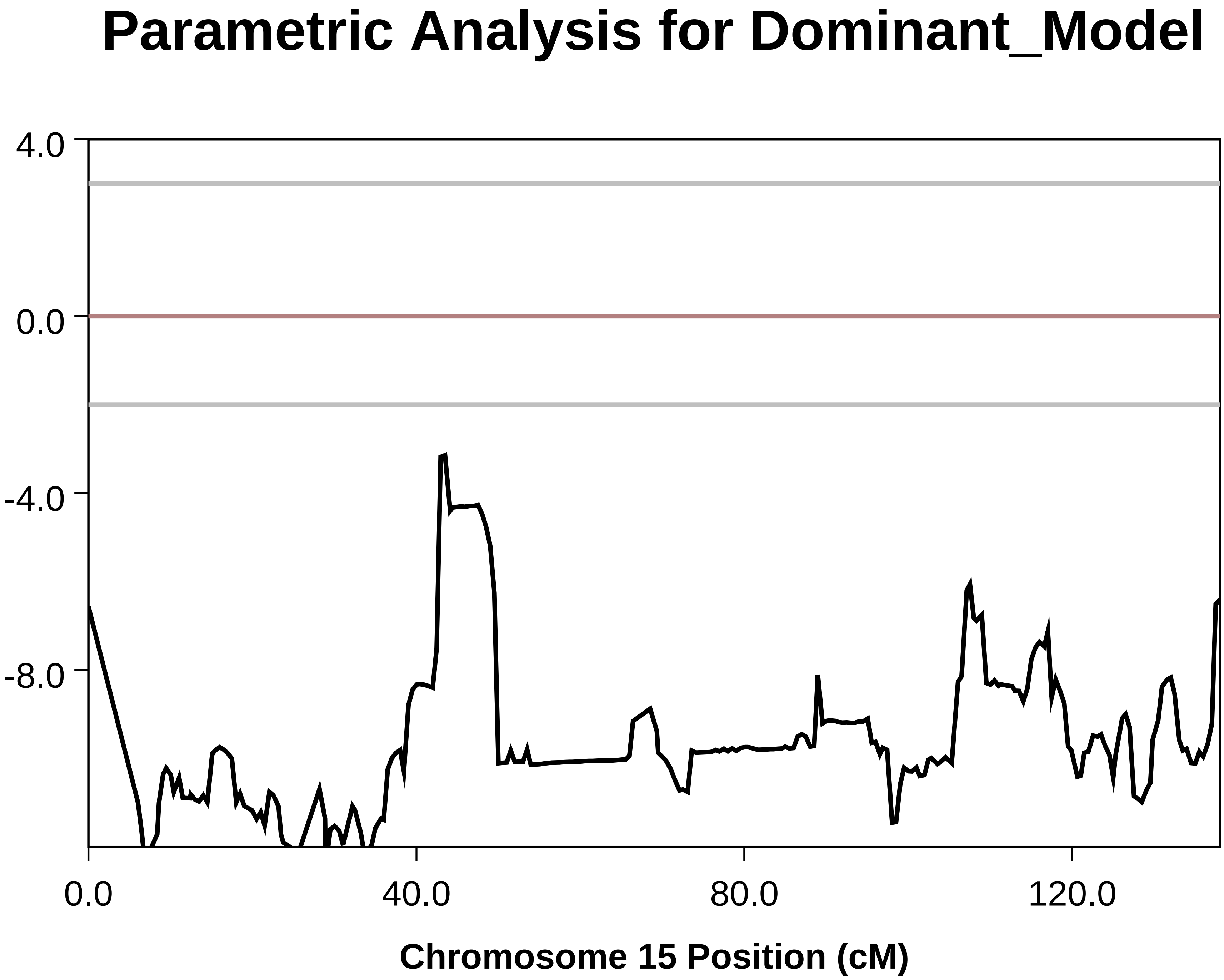
Parametric Analysis for Dominant_Model
4.0
0.0
-4.0
-8.0
0.0
40.0
80.0
120.0
Chromosome 15 Position (cM)

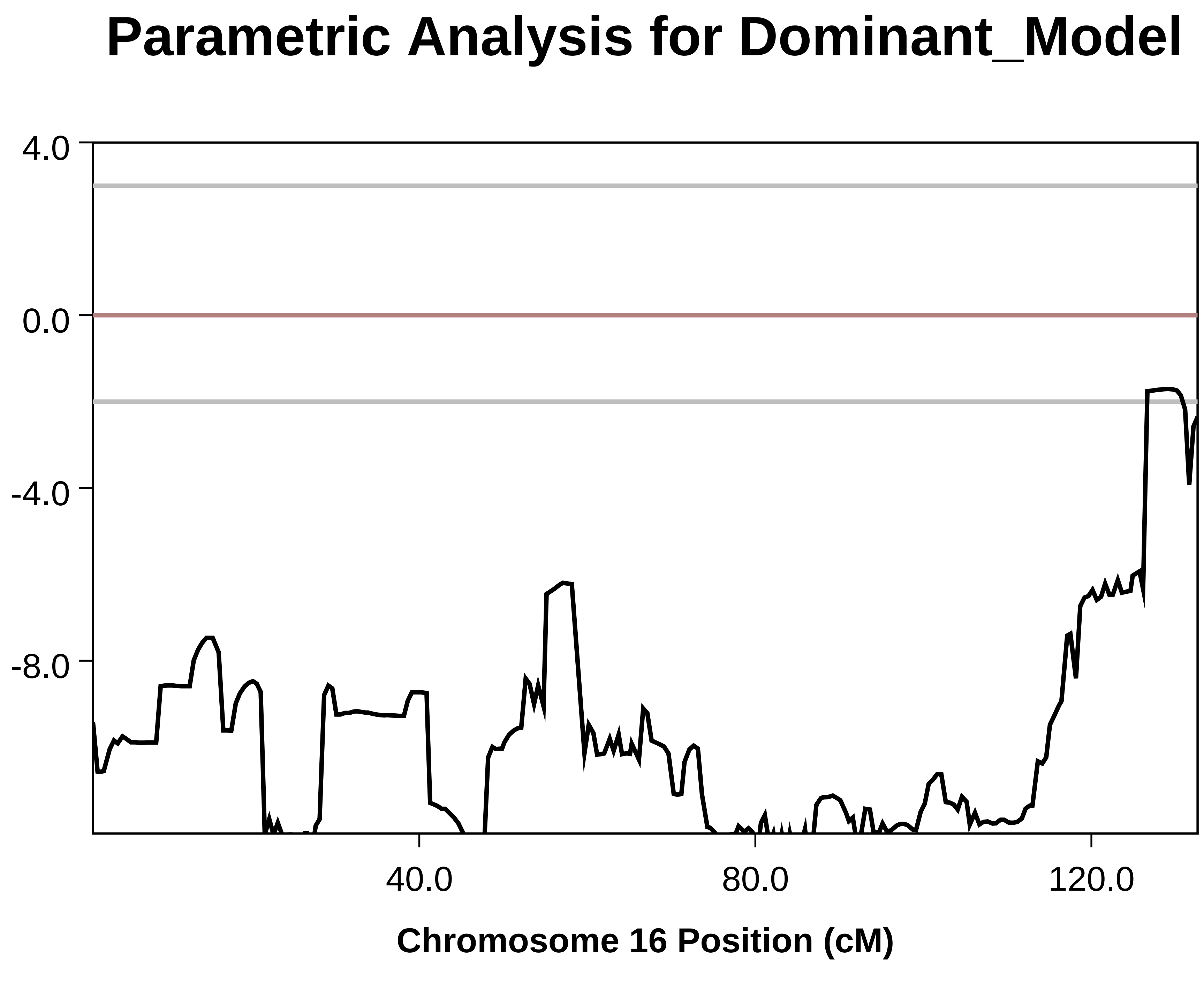

Parametric Analysis for Dominant_Model
4.0
0.0
-4.0
-8.0
40.0
80.0
120.0
Chromosome 16 Position (cM)

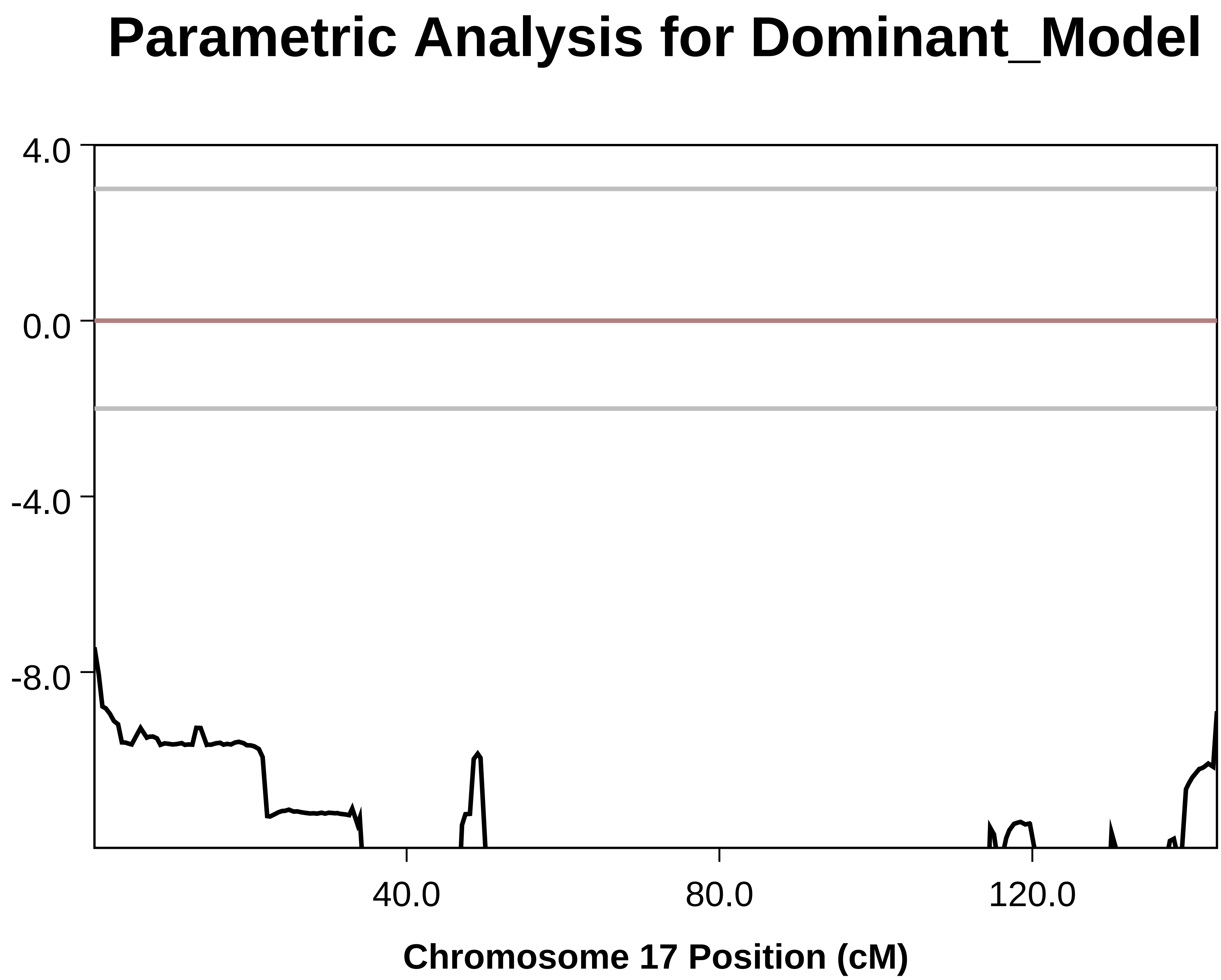

Parametric Analysis for Dominant_Model
4.0
0.0
-4.0
-8.0
40.0
80.0
120.0
Chromosome 17 Position (cM)

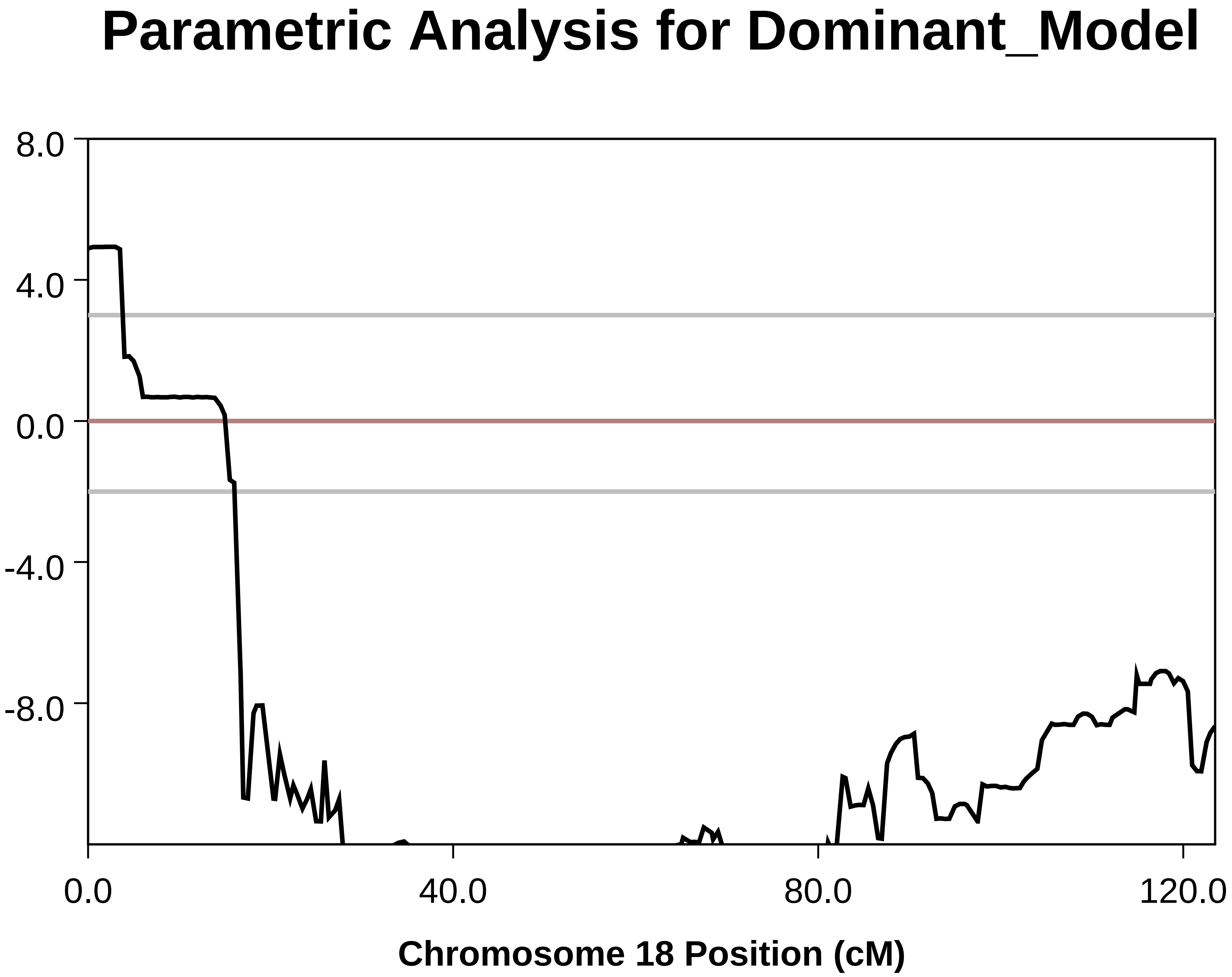
Parametric Analysis for Dominant_Model
8.0
4.0
0.0
-4.0
-8.0
0.0
40.0
80.0
120.0
Chromosome 18 Position (cM)

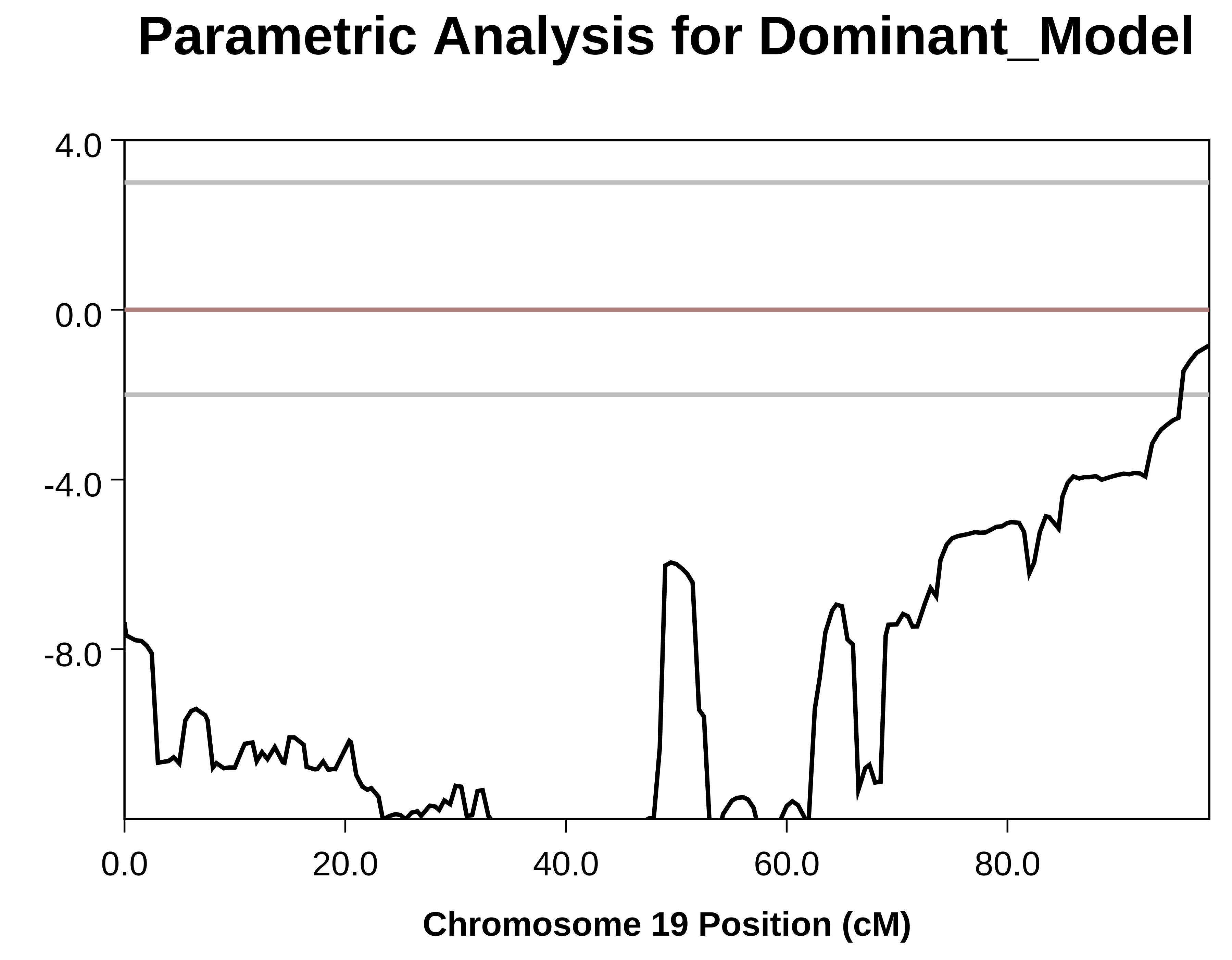
Parametric Analysis for Dominant_Model
4.0
0.0
-4.0
-8.0
0.0
20.0
40.0
60.0
80.0
Chromosome 19 Position (cM)

Parametric Analysis for Dominant_Model

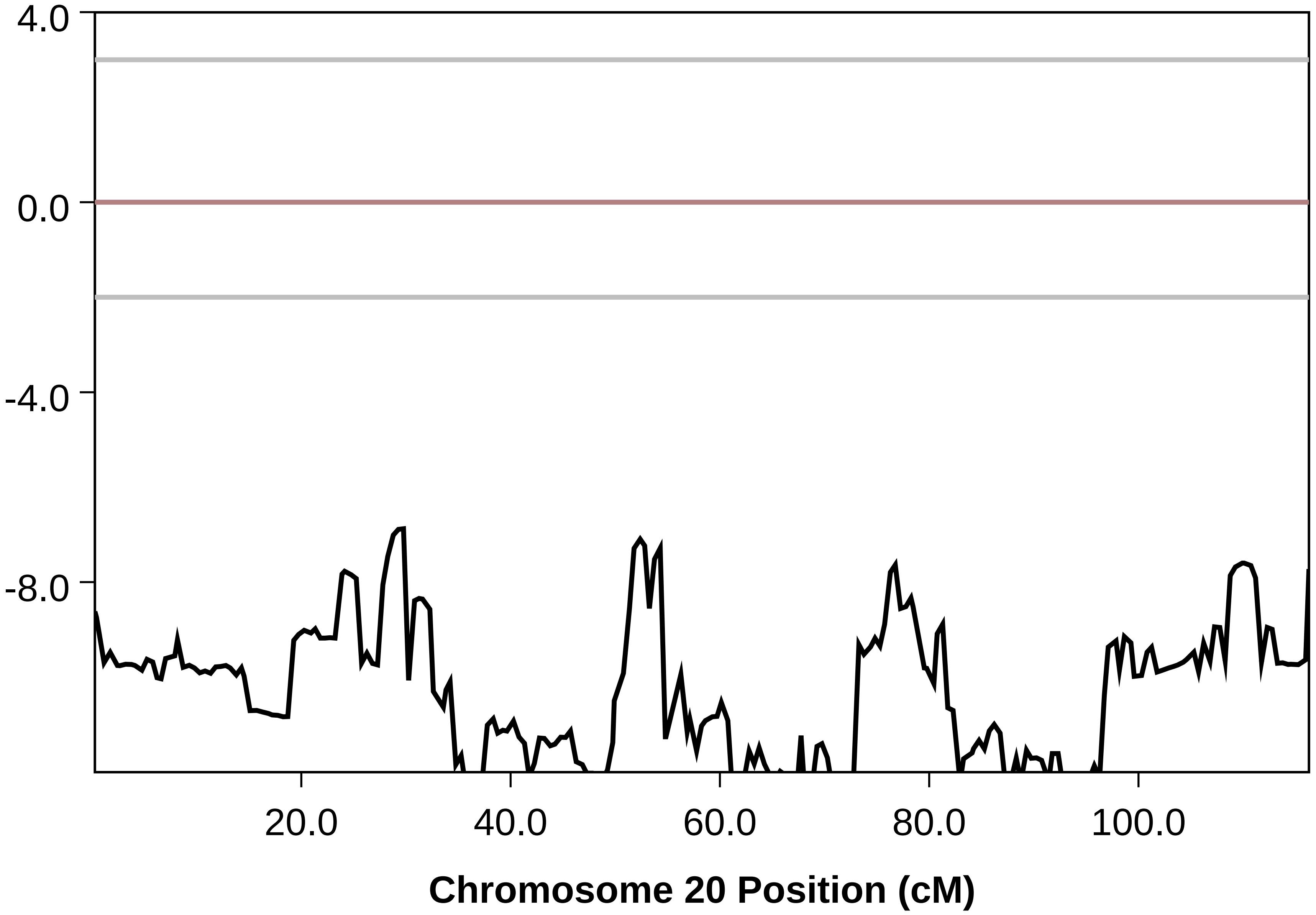

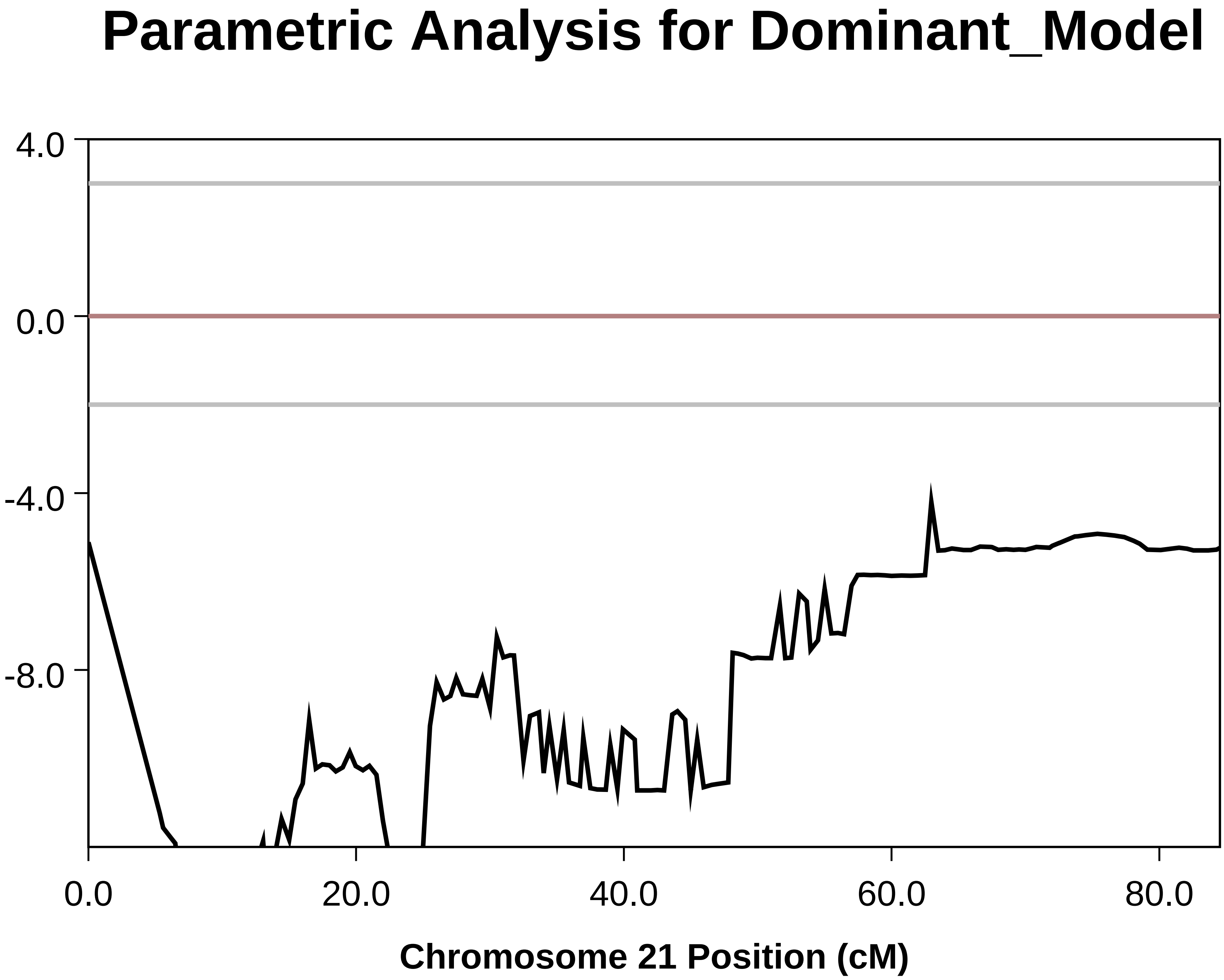

Parametric Analysis for Dominant_Model
4.0
0.0
-4.0
-8.0
0.0
20.0
40.0
60.0
80.0
Chromosome 21 Position (cM)

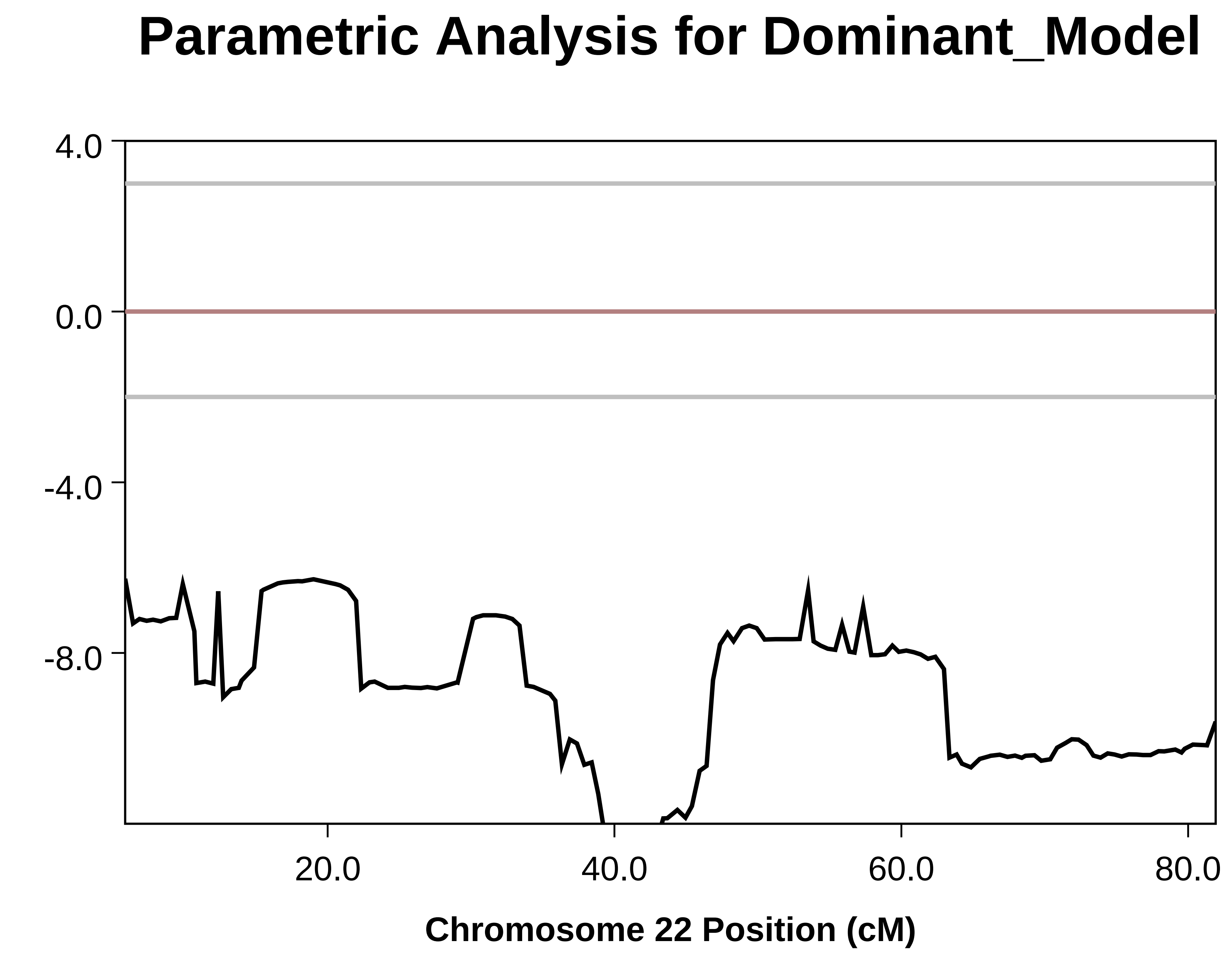

Parametric Analysis for Dominant_Model
4.0
0.0
-4.0
-8.0
20.0
40.0
60.0
80.0
Chromosome 22 Position (cM)
